# Infections and antibiotic use in early childhood have limited importance in developing manifest type 1 diabetes – The ABIS cohort study

**DOI:** 10.3389/fendo.2025.1529447

**Published:** 2025-02-21

**Authors:** Malin Bélteky, Jeanette Wahlberg, Johnny Ludvigsson

**Affiliations:** ^1^ Crown Princess Victoria’s Children´s Hospital, Region Östergötland and Department of Biomedical and Clinical Sciences, Linköping University, Linköping, Sweden; ^2^ Department of Acute Internal Medicine and Geriatrics in Linköping, Department of Health, Medicine and Caring Sciences, Linköping University, Linköping, Sweden; ^3^ Faculty of Medical Sciences, Örebro University, Örebro, Sweden

**Keywords:** childhood environmental factors, gastroenteritis, infections, sex differences, type 1 diabetes

## Abstract

**Aims:**

To investigate the effect of early childhood infections and antibiotic use on the risk of type 1 diabetes in a general population cohort.

**Research Design and Methods:**

The All Babies In Southeast Sweden (ABIS) cohort followed 16 428 children from birth. Questionnaires collected at 1 year (n=11 093), 3 years (n=8 890) and 5 years of age (n=7 445) included data on infections and antibiotic use and were validated against national registers. After a mean follow-up of 25 years, 168 individuals have been diagnosed with type 1 diabetes (1.0% of the original cohort, aged 1-24.5 years).

**Results:**

There were few significant differences in type or frequency of early childhood infections or antibiotic use between cases with type 1 diabetes and the reference group (remaining individuals who did not develop type 1 diabetes) after adjusting for sex, heredity and socioeconomic status. A small number of type 1 diabetes children (4.8% compared to 0.8% of the reference group) reported six or more episodes of gastroenteritis in the 1-3-year age group, resulting in an adjusted odds ratio (aOR) of 8.21; 95% CI 2.70-25.01, p<0.001. Cases of type 1 diabetes with an increased genetic risk (n=91) reported fewer episodes of the common cold between 1 and 3 years of age compared to the reference group (aOR 0.27; 0.13-0.58, p<0.001). Individuals with type 1 diabetes without risk-associated HLA alleles (n=14) reported a higher frequency of pneumonia in the 1–3- and 3–5-year age group (aOR 26.08; 6.29-108.17, p<0.001 and aOR 35.63; 4.10-309.96, p=0.001 respectively), and had more viral and total infections registered in the National Patient Register from 0-5 years (aOR 5.72; 1.59-20.57, p=0.008 and aOR 18.71; 1.95-179.55, p=0.01).

**Conclusions:**

Childhood infections could increase the risk of developing type 1 diabetes in a small group of individuals without risk-associated HLA alleles, but this was not seen in the majority with HLA-risk. More research is required for this overlooked population, including screening and prevention trials. The association to frequent gastrointestinal infections in the first years of life needs to be reproduced in other studies to be confirmed.

## Introduction

The incidence of type 1 diabetes has increased greatly over the past 40-50 years (1), with a peak in global incidence around 10-14 years of age (2). The genetic risk of type 1 diabetes is mainly due to HLA class II genotypes but the incidence of type 1 diabetes in individuals without high HLA-risk has increased (3), and populations of similar genetic backgrounds like Finland and Russian Karelia have shown an age-adjusted six-fold incidence gradient (4).

Several large, prospective cohort studies designed to study environmental risk factors have not found any conclusive evidence, but early exposures like diet (5) and psychosocial stress (6–8) have been suggested. Accumulating evidence points towards a possible interaction between early childhood infections and risk of type 1 diabetes. Prospective studies of mainly genetically at-risk children have found evidence of respiratory tract infections caused by enteroviruses (9, 10) and more recently SARS-CoV-2 (11) preceding onset of overt type 1 diabetes. Gastroenteritis requiring hospitalization during the first 18 months of life has also been reported as a risk factor (12). Viruses have been isolated from the pancreas of patients with new-onset type 1 diabetes (13, 14). We have previously found that respiratory tract infection in early pregnancy increases the risk of childhood type 1 diabetes in the general population (15), and enterovirus infection during pregnancy has been linked to increased risk of childhood-onset type 1 diabetes (16) indicating development of autoimmunity after exposure to viral antigens *in utero*. The “fertile field” hypothesis suggests that repeated infections in childhood create a proinflammatory cytokine environment that could activate autoreactive T-cells in genetically predisposed individuals (17).

The gut microbiome plays an important role in immune system maturation, and the interaction between potential environmental risk factors like diet, breastfeeding (18) and antibiotic use (19), and the activation of autoreactive immune cells through the important barrier function of the gut and the microbiome composition itself is complex (20). In fact, antibiotics could be an important mediator between environmental risks and autoimmunity as it is both associated with alterations in a healthy gut microbiome, as well as a marker for primarily bacterial infections. For instance, early-life exposures to antibiotic treatment has been linked to other diseases like juvenile idiopathic arthritis (21), asthma (22), and neurodevelopmental disorders like autism and ADHD (23). The “hygiene hypothesis” proposes that a decreased microbial load in the environment could trigger the maturing immune system to overreact to otherwise harmless microorganisms (24), while simultaneously decreasing the herd immunity for other potentially harmful microbes (25). Increased hygiene also affects the gut microbiota (26). In a recent study, we found an association between gut microbiome composition in early childhood and development of type 1 diabetes later in life (27), and we have previously described an association between genetic risk for autoimmunity and microbiome composition at 1 year of age (28). As most observational studies only include children with an increased genetic risk, knowledge about individuals without risk-associated HLA alleles is lacking. We hypothesize that gastrointestinal infections and use of antibiotics early in life might play a special role for later development of type 1 diabetes, and that a possible association between childhood infection and type 1 diabetes could be modified by genetic risk of autoimmunity.

The aim of this study was therefore to investigate if type and frequency of childhood infections, especially gastrointestinal infections (“gastroenteritis”), and antibiotic use could affect the risk of type 1 diabetes in a general non-selected population cohort.

## Research design and methods

### Subjects

**Figure 1:**
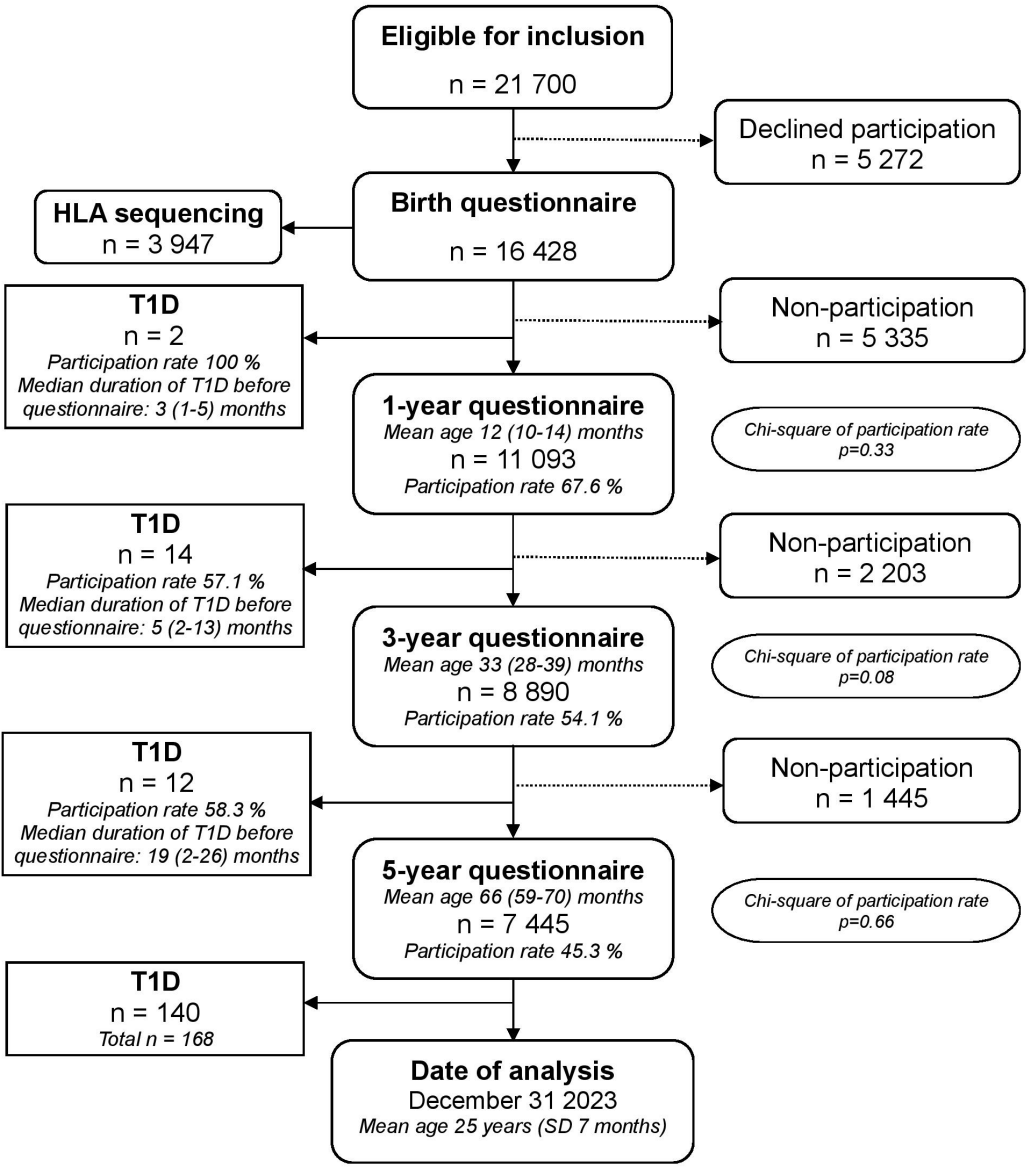
Flow chart of the ABIS birth cohort and participation in the 1-, 3- and 5-year questionnaires. All families participating in the birth questionnaire were invited to participate in all subsequent follow-ups, regardless of participation in previous questionnaires. Mean age at each questionnaire include 95 % confidence intervals. Median duration of type 1 diabetes before participation in the following questionnaire include range in months. Chi-square tests were used to compare participation rates for type 1 diabetes and reference group for each questionnaire, p-values for each questionnaire are included in the flow chart.

All data was derived from the large prospective population-based cohort study All Babies in Southeast Sweden (ABIS). ABIS included children born in Southeast Sweden from October 1^st^ 1997 to October 1^st^ 1999 and they have been followed from birth until adulthood. At birth, the parents of 78.6% of newborn children chose to participate (17 055 of 21 700), of whom we had useful questionnaires from 16 428. In addition to extensive questionnaires, biological samples were collected at each follow-up. See flowchart for details (**Figure 1**). Details about the study layout have been published elsewhere (29); see www.abis-studien.se.

### Procedure

For this study, questionnaires collected at 1, 3 and 5 years of age were used. Each questionnaire retrospectively covered the period from the previous questionnaire. The questionnaires were answered by the child’s parents or caregivers and included, but was not restricted to, questions on diet, infections, drugs, physical activity, psychosocial situation, living situation and other exposures early in life. Before participating in the study, carers were informed of the aim to investigate environmental risk factors for immune-mediated diseases, but not of specific hypotheses about frequency of infections and type 1 diabetes.

Carers reported frequency of infections occurring during the period between two questionnaires in intervals of “none”, “1-2”, “3-5” or “6 or more”. Data on specific age at each infection, severity and duration of infections or associated symptoms (i.e. fever) were not available for analysis.

Beside data on infections from the questionnaires, The Swedish National Patient Register (NPR) was used to collect data on infections registered from hospital admissions and specialized outpatient health care visits from birth to 5 years of age for all children enrolled in ABIS. These visits were initiated by the families themselves and represent acute infections. The NPR data was restricted to date of visit and ICD-10 diagnostic codes; information on duration and associated symptoms were not available in the NPR. The NPR does not include data from primary health care visits. The ICD-10 diagnostic codes were used to manually categorize infections into respiratory tract infections, gastroenteritis, urinary tract infection, and unspecified viral infections. All infections from the questionnaires and the NPR were further categorized into either bacterial or viral according to most likely infectious agent, i.e. influenza was categorized as viral while pneumonia, acute otitis media and urinary tract infection were categorized as bacterial. Gastroenteritis was categorized as viral unless otherwise stated in the ICD-10 code. As the reported frequency of the common cold was extremely high for all age groups (>99%), this infection was not included in the analysis of viral infections from the questionnaires. The ICD-10 codes were reviewed to exclude duplicates registered for the same infection dating within 14 days of the first code, i.e. a tonsillitis with a follow-up visit registered within a few days with a similar diagnostic code would count as the same respiratory tract infection both visits. Initial analyses of data from the NPR were performed by combining all registered infections in each category as “1 or more registered infections” compared to “No infections”. If possible, infections were then binned into 3 categories: “No registered infections”, “1-2 registered infections” and “3 or more registered infections”.

A diagnosis of type 1 diabetes was identified via the Swedish National Patient Register (NPR) and The Swedish Childhood Diabetes Registry (SWEDIABKIDS) and validated by the Swedish National Drug Prescription Register for prescription of insulin. Age in months at onset of diabetes was calculated based on date of birth and date of diagnosis as registered in the NPR, SWEDIABKIDS, and/or date of first insulin prescription.

Genetic risk of type 1 diabetes was determined by HLA genotyping and categorized according to presence of common European HLA-DR-DQ haplotypes associated with the risk of autoimmunity (30). Increased or high genetic risk was defined as the presence of one or two risk-associated HLA haplotypes: (DR3)-DQA1*05-DQB1*02 with no protective haplotypes, and/or (DR4)-DQA1*03-DQB1*0302 with or without protective haplotypes. A neutral or low genetic risk was defined as the presence of (DR3)-DQA1*05-DQB1*02 in combination with one of the protective haplotypes or the absence of any risk-associated haplotypes with one or two protective haplotypes: (DR15)-DQB1*0602, (DR13)-DQB1*0603, (DR5)-DQA1*05-DQB1*0301 and (DR7)-DQA1*03-DQB1-0303.

### Statistical methods

The data was stored in a common database and statistically analyzed using the SPSS 29.0 program (IBM Corp., Armonk, NY: USA).

An adjusted odds ratio (aOR) and 95% confidence intervals (CI) were estimated for each explanatory variable by multiple logistic regression analysis, adjusting for interaction with sex, family history of type 1 diabetes and socioeconomic status. Socioeconomic status (SES) defined as maternal education level at birth was included as confounder due to previously reported associations with type 1 diabetes in this cohort (31). Basic characteristics of the cohort were analyzed using Chi^2^ tests.

The primary hypothesis tested was whether exposure to infections or antibiotics during early childhood affected the risk of type 1 diabetes in the general population. The initial analyses were based on age (birth to 1 year, 1 to 3 years, 3 to 5 years), type of infection, likely infectious agent (virus or bacteria) and use of antibiotics. Secondary analyses were performed based on HLA risk group, age at onset of type 1 diabetes, and sex, as boys have previously been described as more susceptible to infections. A cut-off age of 10.5 years for girls and 11.5 years for boys was chosen to distinguish onset of type 1 diabetes from before and after the start of puberty (32), as exposure to early childhood infections is hypothesized to have a greater impact on the progress to overt disease in younger children. In the subgroup analyses based on sex, the variable “sex” was removed as a confounder from the logistic regression. To adjust for multiple testing, the Benjamini & Hochberg procedure was performed to determine significance using a false discovery rate (FDR) of 0.05. Infections reported after onset of type 1 diabetes were excluded. In cases where questionnaires were returned within ≤3 months after diagnosis (n=5), reported infections from that questionnaire were still included in the analyses due to the inability to pinpoint exact timing of infections in relation to onset of disease, and risk of losing valuable information about the period preceding overt type 1 diabetes.

### Ethical consideration

Written and oral information about the project was given to the parents already during pregnancy, and then repeated at the maternity ward after birth and at the well-baby checkups. Participating families were also offered the opportunity to watch a video about the study. Informed consent was given when the parents or guardians returned the questionnaires at birth, 1, 3 or 5 years or delivered biological samples.

The ABIS project was approved by the Research Ethics Committees of the Faculty of Health Science at the University of Linköping, Linköping, Sweden (Dnr-96-287, Dnr-99-321 and Dnr-03-092) and the Medical Faculty at the University of Lund, Lund, Sweden (LU 83-97). Linking the ABIS registers to the NPR was approved by the Research Ethics Committee in Linköping (Dnr-03-513 and Dnr-2018/380-32).

### Data and resource availability

The datasets generated and/or analyzed during the current study are available from the last author on reasonable request after ethical approval.

## Results

### Characteristics of the ABIS birth cohort

As of December 31st 2023, 168 individuals from the original cohort had developed type 1 diabetes (1.0%), 56.0% males and 44.0% females compared to 51.8% males and 48.2% females in the reference group without type 1 diabetes. With a mean follow-up of 25 years 2 months (SD 7 months), 168 cases of type 1 diabetes correspond to an incidence rate of 40.9/100 000 individuals/year. HLA genotyping data was available for 3947 study participants. In the HLA-typed group with type 1 diabetes (n=105), 86.7% were categorized as having a high or increased genetic risk, compared to 38.3% of the reference group (p<0.001). Having a first-degree relative with type 1 diabetes was associated with an 11-fold increased risk of developing type 1 diabetes (OR 11.47; 95% CI 6.78-19.40, p<0.001). Low and intermediate maternal education at birth was associated with an increased risk of type 1 diabetes compared to high maternal education, defined as International Standard Classification of Education (ISCED) level I-IV compared to level V-VII (**Table 1**).

**Table 1:** Characteristics of the ABIS cohort.

	Type 1 diabetes n (%)	Reference group n (%)	p-value
Sex			0.28
Males	94 (56.0)	8390 (51.8)	
Females	74 (44.0)	7808 (48.2)	
Family with type 1 diabetes	17 (10.1)	158 (1.0)	**<0.001**
Maternal education			**0.02**
Low and intermediate	126 (76.8)	10775 (68.2)	
High	38 (23.2)	5030 (31.8)	
HLA risk			**<0.001**
High and increased	91 (86.7)	1470 (38.3)	
Neutral and decreased	14 (13.3)	2372 (61.7)	

Basic characteristics of the ABIS cohort analyzed with chi-square tests. Bolded p-values are statistically significant after Benjamini & Hochberg correction for multiple comparisons.

Age at diagnosis of type 1 diabetes ranged from 12 months to 24 years 6 months, with a mean age at diagnosis of 12 years 3 months. Mean age at diagnosis differs almost 1.5 years between females (11 years, 5 months) and males (12 years, 10 months) due to a large overrepresentation of males with an onset of type 1 diabetes after 17 years (75% males). Having a neutral or decreased genetic risk of type 1 diabetes was associated with a mean age at diagnosis 3.5 years later than those with a high or increased genetic risk (13 years, 9 months compared to 10 years, 2 months, **Table 2**).

**Table 2:** Characteristics of cases with type 1 diabetes.

	Total n	Boys n (%)	Girls n (%)	Mean age at diagnosis^a^ (SD)	Boys mean age^a^ (SD)	Girls mean age^a^ (SD)	Range^b^
All T1D	168	94 (56.0)	74 (44.0)	12.3 (6.2)	12.8 (6.5)	11.6 (5.8)	1-24.5
Before puberty	72	39 (54.2)	33 (45.8)	6.4 (3.0)	6.5 (3.2)	6.4 (2.8)	1-10.5
After puberty	96	55 (57.3)	41 (42.7)	16.7 (3.9)	17.3(3.9)	15.8 (3.8)	10.6-24.5
High and increased risk	91	56 (61.5)	35 (38.5)	10.2 (6.0)	10.5 (6.3)	9.6 (5.4)	1-23.5
Neutral and decreased risk	14	9 (64.3)	5 (35.7)	13.8 (4.0)	13.6 (4.7)	14.0 (5.0)	6.3-19.2

^a^Mean age at diagnosis in years. ^b^Age range at diagnosis in years.

### Infections during the first five years of life

**Figure 2:**
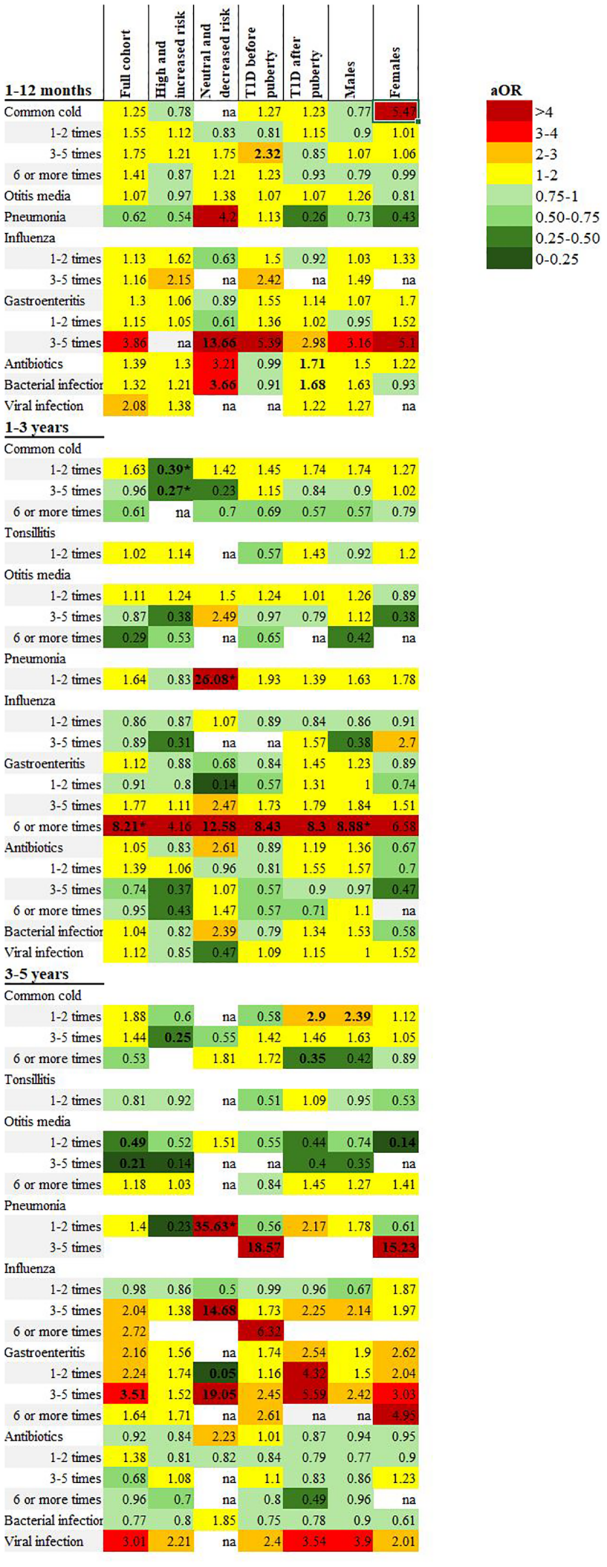
Heatmap illustrating adjusted odds ratios of associations between early childhood infections reported in the 1-, 3- and 5-year questionnaires and subgroup analyses including the full cohort, high and increased genetic risk, neutral and decreased genetic risk, onset of type 1 diabetes before and after puberty and males/females. All included analyses were adjusted for sex, family history of type 1 diabetes and maternal education. Bolded text indicates CI below/above 1. *Statistically significant after Benjamini & Hochberg correction for multiple comparisons.

The common cold was by far the most common infection in all age categories with no difference in frequency between cases of type 1 diabetes and the reference group (**Table 3**). The other respiratory tract infections analyzed (otitis media, tonsillitis, pneumonia, influenza) did not differ between the groups (**Supplementary Table 1**). Infections primarily interpreted as either bacterial or viral were reported similarly for both cases of type 1 diabetes and the reference group for all age groups, and there were no significant differences in reported number of antibiotic treatments between the groups (**Table 3**, **Figure 2**).

**Table 3:** Infections during the first 5 years of life in individuals with type 1 diabetes compared to a reference group without type 1 diabetes.

	Type 1 diabetes n (%)	Reference group n (%)	Adj. OR^a^ (95 % CI)	Adj. p-value^a^
**Total**	168	16260		
1-12 months				
Common cold				
1-2 times	32 (31.4)	3515 (33.9)	1.55 (0.21-11.46)	0.67
3-5 times	54 (52.9)	4988 (48.1)	1.75 (0.24-12.79)	0.58
6 or more times	15 (14.7)	1702 (16.4)	1.41 (0.18-10.83)	0.74
Otitis media	27 (27.6)	2581 (26.4)	1.07 (0.68-1.68)	0.77
Pneumonia	4 (4.3)	611 (6.6)	0.62 (0.23-1.70)	0.35
Influenza				
1-2 times	12 (13.5)	1122 (12.7)	1.13 (0.61-2.10)	0.69
3-5 times	1 (1.1)	58 (0.7)	1.16 (0.15-9.20)	0.89
Gastroenteritis	35 (34.7)	3011 (29.4)	1.30 (0.85-1.96)	0.22
1-2 times	29 (32.2)	2759 (30.0)	1.15 (0.73-1.80)	0.56
3-5 times	2 (2.2)	72 (0.8)	3.86 (0.92-16.21)	0.07
Antibiotics	44 (47.3)	3788 (39.7)	1.39 (0.92-2.11)	0.12
Bacterial infection	46 (44.7)	4030 (38.4)	1.32 (0.89-1.96)	0.17
Viral infection	106 (99.1)	10654 (98.0)	2.08 (0.29-15.02)	0.47
1 to 3 years				
Common cold				
1-2 times	16 (18.4)	968 (11.2)	1.63 (0.87-3.04)	0.13
3-5 times	37 (42.5)	4103 (47.6)	0.96 (0.60-1.56)	0.88
6 or more times	34 (39.1)	3530 (41.0)	0.61 (0.33-1.15)	0.12
Tonsillitis				
1-2 times	11 (14.1)	1142 (14.7)	1.02 (0.53-1.95)	0.96
Otitis media				
1-2 times	36 (41.4)	3060 (37.9)	1.11 (0.70-1.77)	0.65
3-5 times	8 (9.2)	839 (10.4)	0.87 (0.40-1.89)	0.73
6 or more times	1 (1.1)	285 (3.5)	0.29 (0.04-2.14)	0.22
Pneumonia				
1-2 times	10 (12.5)	556 (7.1)	1.64 (0.81-3.34)	0.17
Influenza				
1-2 times	26 (33.3)	2727 (35.2)	0.86 (0.53-1.41)	0.55
3-5 times	3 (3.8)	342 (4.4)	0.89 (0.27-2.90)	0.85
Gastroenteritis	63 (75.0)	6062 (73.4)	1.12 (0.67-1.87)	0.67
1-2 times	41 (49.4)	5028 (60.9)	0.91 (0.53-1.56)	0.72
3-5 times	17 (20.5)	956 (11.6)	1.77 (0.90-3.51)	0.1
6 or more times	4 (4.8)	65 (0.8)	8.21 (2.70-25.01)	**<0.001**
Antibiotics	60 (67.4)	5783 (66.6)	1.05 (0.66-1.66)	0.83
1-2 times	40 (49.4)	3381 (42.2)	1.39 (0.85-2.28)	0.19
3-5 times	9 (11.1)	1108 (13.8)	0.74 (0.33-1.66)	0.47
6 or more times	3 (3.7)	317 (4.0)	0.95 (0.28-3.20)	0.95
Bacterial infection	60 (69.8)	5814 (69.5)	1.04 (0.65-1.68)	0.87
Viral infection	76 (87.4)	7170 (85.3)	1.12 (0.59-2.13)	0.73
3 to 5 years				
Common cold				
1-2 times	7 (10.1)	511 (7.1)	1.88 (0.80-4.41)	0.15
3-5 times	37 (53.6)	3218 (44.5)	1.44 (0.85-2.44)	0.17
6 or more times	25 (36.2)	3491 (48.3)	0.53 (0.23-1.26)	0.15
Tonsillitis				
1-2 times	11 (17.5)	1378 (19.9)	0.81 (0.41-1.62)	0.56
Otitis media				
1-2 times	16 (25.0)	2533 (36.3)	0.49 (0.27-0.90)	0.02
3-5 times	2 (3.1)	711 (10.2)	0.21 (0.05-0.89)	0.03
6 or more times	3 (4.7)	189 (2.7)	1.18 (0.35-3.93)	0.79
Pneumonia				
1-2 times	5 (7.9)	360 (5.2)	1.40 (0.55-3.58)	0.49
Influenza				
1-2 times	24 (36.4)	2720 (39.7)	0.98 (0.57-1.69)	0.93
3-5 times	8 (12.1)	459 (6.7)	2.04 (0.92-4.53)	0.08
6 or more times	1 (1.5)	50 (0.7)	2.72 (0.36-20.50)	0.33
Gastroenteritis	65 (92.9)	6352 (88.2)	2.16 (0.78-5.99)	0.14
1-2 times	40 (58.0)	4390 (61.8)	2.24 (0.69-7.29)	0.18
3-5 times	24 (34.8)	1768 (24.9)	3.51 (1.05-11.75)	0.04
6 or more times	1 (1.4)	163 (2.3)	1.64 (0.17-16.02)	0.67
Antibiotics	48 (68.6)	5197 (71.1)	0.92 (0.55-1.56)	0.77
1-2 times	33 (53.2)	2989 (43.4)	1.38 (0.79-2.41)	0.26
3-5 times	5 (8.1)	948 (13.8)	0.68 (0.25-1.81)	0.44
6 or more times	2 (3.2)	268 (3.9)	0.96 (0.22-4.15)	0.95
Bacterial infection	46 (68.7)	5240 (73.0)	0.77 (0.45-1.31)	0.33
Viral infection	67 (95.7)	6582 (91.4)	3.01 (0.73-12.37)	0.13
National Patient Register				
Respiratory Tract Infection	24 (14.3)	2481 (15.3)	0.61 (0.30-1.23)	0.16
1-2 times	23 (13.7)	2378 (14.6)	0.64 (0.32-1.29)	0.21
3 or more times	1 (0.6)	103 (0.6)	0.83 (0.11-6.08)	0.86
Gastroenteritis	4 (2.4)	219 (1.3)	1.54 (0.36-6.56)	0.56
Urinary Tract Infection	4 (2.4)	229 (1.4)	2.14 (0.52-8.87)	0.29
Unspecified infection	12 (7.1)	929 (5.7)	1.85 (0.91-3.75)	0.09
Total infection	33 (19.6)	3398 (20.9)	0.79 (0.46-1.37)	0.41
1-2 times	27 (16.1)	3126 (19.2)	0.74 (0.42-1.33)	0.32
3 or more times	6 (3.6)	272 (1.7)	1.48 (0.35-6.20)	0.6
Bacterial infection	13 (7.7)	1442 (8.9)	0.80 (0.35-1.86)	0.61
1-2 times	12 (7.1)	1414 (8.7)	0.82 (0.35-1.89)	0.64
3 or more times	1 (0.6)	28 (0.2)	3.75 (0.51-27.75)	0.20
Viral infection	30 (17.9)	2421 (14.9)	1.09 (0.62-1.92)	0.77
1-2 times	29 (17.3)	2331 (14.3)	1.13 (0.64-1.99)	0.68
3 or more times	1 (0.6)	90 (0.6)	1.08 (0.15-7.91)	0.94

Logistic regression analysis adjusting for sex, family history of type 1 diabetes and maternal education. Bolded p-values are considered statistically significant after Benjamini & Hochberg correction for multiple comparisons.

Six or more episodes of gastroenteritis in the 1-3-year age group was reported for 4.8% of children with type 1 diabetes compared to 0.8% of the reference group (adjusted odds ratio (aOR) 8.21; 2.70-25.01, p<0.001). Of the children with a diagnosis of type 1 diabetes before five years of age (n=26), half (n=13) had reported one or more episodes of gastroenteritis after the onset of diabetes. These episodes were excluded from the analyses.

### Infections from the national patient register

**Figure 3:**
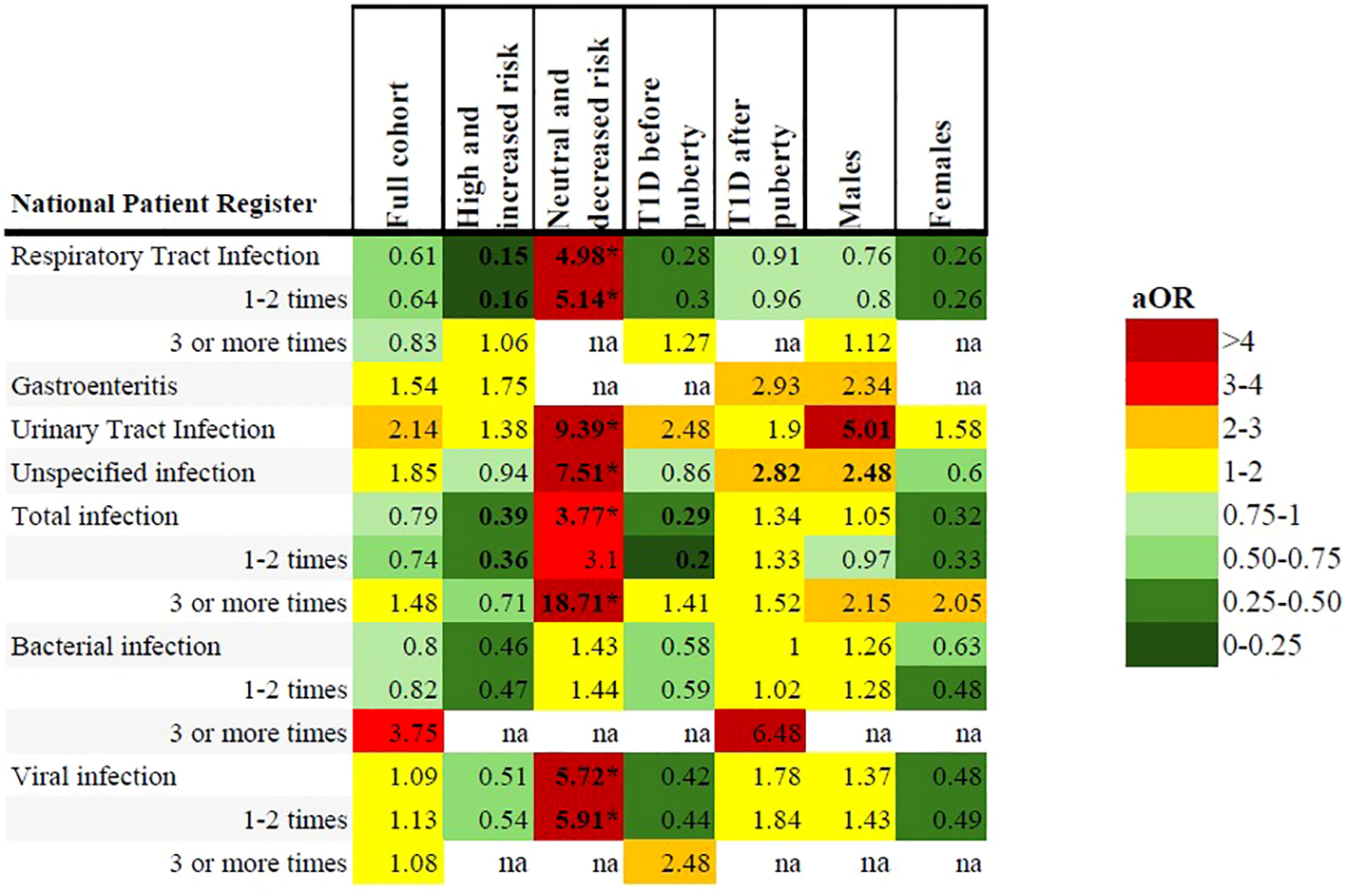
Heatmap illustrating adjusted odds ratios of associations between early childhood infections registered in the NPR and subgroup analyses including the full cohort, high and increased genetic risk, neutral and decreased genetic risk, onset of type 1 diabetes before and after puberty and males/females. All included analyses were adjusted for sex, family history of type 1 diabetes and maternal education. Bolded text indicates CI below/above 1. *Statistically significant after Benjamini & Hochberg correction for multiple comparisons.

Most recorded infections from the NPR in the first five years of life were categorized as respiratory tract infections (66.8%), with unspecified viral infections the second most common (22.1%). The total number of registered infections was lower than the reported number of infections in the questionnaires. Only 20.9% of ABIS children had one or more registered infections from ages 0-5 years in the NPR. Of these, 14.9% had infections categorized as viral and 8.9% had infections categorized as bacterial. There was no statistically significant difference in frequency of any type of infection between the ages of 0-5 for the cohort (**Table 3**, **Figure 3**).

### High or increased genetic risk of type 1 diabetes

Type 1 diabetes cases with a high or increased genetic risk (n=91, 86.7%) had significantly fewer episodes of the common cold compared to the reference group with the same genetic risk (n=1470, 38.3%). In the 1-3-year age group, 3-5 episodes of the common cold was reported for 31.3% of children with type 1 diabetes compared to 44.5% of the reference group (aOR 0.27; 0.13-0.58, p<0.001). There were no other significant associations with infections from the questionnaires.

In the NPR, respiratory tract infections were registered less frequently in the type 1 diabetes group (11.0% compared to 16.2% of the reference group), but this was not statistically significant after correcting for multiple comparisons, (**Table 4**, **Figures 2** and **3** and **Supplementary Table 2**).

**Table 4:** Significant associations with infections in children with a diagnosis of type 1 diabetes from the subgroup analyses based on genetic risk of autoimmunity, onset of type 1 diabetes before or after puberty and sex.

	Type 1 diabetes n (%)	Reference group n (%)	Adjusted OR (95 % CI)	p-value
**High and increased risk**	91	1470		
*1-3 years*				
Common cold				
1-2 times	28 (45.9)	510 (46.0)	0.39 (0.19-0.80)	**0.01**
3-5 times	19 (31.1)	494 (44.5)	0.27 (0.13-0.58)	**<0.001**
*3-5 years*				
Common cold				
1-2 times	29 (58.0)	393 41.9)	0.60 (0.23-1.54)	0.28
3-5 times	15 (30.0)	482 (51.4)	0.25 (0.09-0.71)	0.009
*Patient Register*				
Respiratory tract infection	10 (11.0)	238 (16.2)	0.15 (0.03-0.62)	0.009
1-2 times	9 (9.9)	227 (15.4)	0.16 (0.04-0.67)	0.01
**Neutral and decreased risk**	14	2372		
*1-12 months*				
Bacterial infection	9 (69.2)	744 (38.3)	3.66 (1.10-12.13)	0.03
*1-3 years*				
Pneumonia	5 (55.6)	88 (5.4)	26.08 (6.29-108.17)	**<0.001**
*3-5 years*				
Pneumonia	2 (40.0)	60 (4.1)	35.63 (4.10-309.96)	**0.001**
*Patient Register*				
Respiratory tract infection	5 (35.7)	343 (14.5)	4.98 (1.31-18.95)	**0.02**
Urinary tract infection	1 (7.1)	26 (1.1)	9.39 (1.05-83.77)	**0.05**
Unspecified infection	3 (21.4)	135 (5.7)	7.51 (1.81-31.11)	**0.005**
Total infection	6 (42.9)	486 (20.5)	3.77 (1.06-13.47)	**0.04**
1-2 times	5 (35.7)	455 (19.2)	3.10 (0.80-12.02)	0.1
3 or more times	1 (7.1)	31 (1.3)	18.71 (1.95-179.55)	**0.01**
Viral infection	6 (42.9)	355 (15.0)	5.72 (1.59-20.57)	**0.008**
**Debut before puberty**	72			
*1-3 years*				
Gastroenteritis	26 (70.3)	6062 (73.4)	0.84 (0.41-1.72)	0.63
1-2 times	14 (38.9)	5028 (60.9)	0.57 (0.25-1.26)	0.16
3-5 times	9 (25.0)	956 (11.6)	1.73 (0.68-4.39)	0.25
6 or more times	2 (5.6)	65 (0.8)	8.43 (1.79-39.66)	0.007
*3-5 years*				
Pneumonia				
1-2 times	1 (3.6)	360 (5.2)	0.56 (0.07-4.21)	0.57
3-5 times	1 (3.6)	19 (0.3)	18.57 (2.35-146.88)	0.006
**Debut after puberty**	96			
*1-12 months*				
Antibiotics	30 (51.7)	3788 (39.7)	1.71 (1.01-2.90)	0.04
Bacterial infection	31 (50.0)	4030 (38.4)	1.68 (1.01-2.79)	0.04
*1-3 years*				
Gastroenteritis	37 (78.7)	6062 (73.4)	1.45 (0.69-3.01)	0.33
1-2 times	27 (57.4)	5028 (60.9)	1.31 (0.61-2.80)	0.48
3-5 times	5 (10.2)	956 (11.6)	1.79 (0.66-4.83)	0.25
6 or more times	2 (4.3)	65 (0.8)	8.30 (1.74-39.50)	0.008
**Boys**	94	8390		
*1-3 years*				
Gastroenteritis	43 (72.9)	3133 (73.6)	1.23 (0.66-2.31)	0.52
1-2 times	29 (51.8)	2591 (60.9)	1.00 (0.51-1.94)	0.99
3-5 times	10 (17.9)	500 (11.7)	1.84 (0.80-4.27)	0.15
6 or more times	3 (5.4)	36 (0.8)	8.88 (2.39-32.96)	**0.001**

Logistic regression analysis adjusting for sex, family history of type 1 diabetes and maternal education, each subgroup was analyzed separately. Analysis of sex differences did not include sex as a confounder in the logistic regression. Bolded p-values are considered statistically significant after Benjamini & Hochberg correction for multiple comparisons.

### Neutral or decreased genetic risk of type 1 diabetes

In the type 1 diabetes group, 14 individuals had a neutral or decreased genetic risk of autoimmunity (13.3%), compared to 2372 of the reference group (61.7%). Pneumonia was reported more frequently for the type 1 diabetes group in all three age categories, statistically significant in the 1-3- and 3-5-year age group (aOR 26.08; 6.29-108.17, p<0.001, and 35.63; 4.10-309.96, p=0.001, respectively). In the 1-12-month age group, both antibiotics and bacterial infections were more frequently reported in the type 1 diabetes group (69.2% compared to 39.3%), though this association was not statistically significant.

In the NPR, children with type 1 diabetes had significantly more registered respiratory tract infections, urinary tract infections, unspecified infections, viral infections and total infections compared to the reference group. A total of three or more infections was associated with an aOR of 18.71 (1.95-179.55), p=0.01 (**Table 4**, **Figures 2**, **3**, **Supplementary Table 3**).

### Onset of type 1 diabetes before puberty

A diagnosis of type 1 diabetes before puberty (n=72) was associated with both a self-reported six or more episodes of gastroenteritis in the 1-3 year age group (aOR 8.43; 1.79-39.66, p=0.007), and 3-5 episodes of pneumonia in the 3-5 year age group (aOR 18.57; 2.35-146.88, p=0.006) but neither association was significant after correcting for multiple comparisons and are likely due to small sample sizes. There were no other statistically significant differences in either the questionnaires nor the NPR for any other type of infection or antibiotic treatment (**Table 3**, **Figures 2**, **3**, **Supplementary Table 4**).

### Onset of type 1 diabetes after puberty

Cases with an onset of type 1 diabetes after puberty (n=96) had an association with six or more episodes of gastroenteritis in the 1-3-year age group (aOR 8.30; 1.74-39.50, p=0.008), but this association was not significant after correcting for multiple comparisons. Antibiotics and infections categorized as bacterial were more frequently reported for the type 1 diabetes group (51.7% vs 39.7%, and 50.0% vs 38.4% respectively) in the 0-1-year age group, but this difference was also not statistically significant (**Table 4**, **Figure 2**, **Supplementary Table 5**).

There were no statistically significant associations with any registered infections in the NPR (**Figure 3**, **Supplementary Table 5**).

### Sex differences

Males with type 1 diabetes (n=94) had a significant association with six or more episodes of gastroenteritis in the 1-3-year age group (reported for 5.4% of males with type 1 diabetes compared to 0.8% of reference males, aOR 8.88; 2.39-32.96, p=0.001). There were no significant associations with any other types of infection in either the questionnaires or the NPR (**Table 4**, **Figures 2**, **3**, **Supplementary Table 6**).

Females with type 1 diabetes (n=74) had no significant association with any infection in the questionnaires or the NPR (**Supplementary Table 7**).

## Discussion

In general, there were no significant differences in type or frequency of reported infections in the ABIS questionnaires between cases with type 1 diabetes and the reference group without type 1 diabetes during the observed period of 0-5 years of age. However, in the 1-3-year age group, a reported six or more episodes of gastroenteritis were significantly associated with type 1 diabetes. This correlates with earlier findings of the importance of the microbiome at one year of age (27) and could represent a pathophysiological mechanism linking gastrointestinal infections and autoimmunity through an altered gut microbiome in early childhood. Instances of gastroenteritis reported after a diagnosis of type 1 diabetes were excluded from the analyses, mitigating the risk of a false association caused by an increased susceptibility of infections in patients with dysglycemia. It is important to note that only 4 children with type 1 diabetes reported 6 or more episodes of gastroenteritis in this age group. To determine the significance of this association, these results need to be replicated in a larger cohort of children with type 1 diabetes. Notably, there were no significant differences in number of antibiotic treatments between the groups for any age category, contradicting the theory of transient gut dysbiosis as an important promotor of the autoimmune process for this cohort. These findings align with results from another questionnaire-based study (12) as well as data from a prospective cohort of genetically at-risk children (33). Other register-based studies have found evidence of antibiotic treatments, especially broad-spectrum (19) or antibiotics prescribed for respiratory tract infections (34), in the first 1-2 years of life to increase the risk of childhood type 1 diabetes in children delivered by cesarean section but not in vaginally delivered children, implying a vulnerability to the gut-altering effects of antibiotics in only certain circumstances. Unfortunately, the analyses in this cohort did not specify use of broad-spectrum or narrow-spectrum antibiotics, and data from the Swedish Drug Register was not available during this period.

Some individuals with type 1 diabetes have no risk-associated HLA alleles, and environmental triggers initiating the autoimmune process in these cases are arguably more consequential. This was reflected in a later diabetes debut compared to children with a high genetic risk. In this group, both viral and bacterial infections, like pneumonia, were significantly associated with type 1 diabetes. The autoimmune process in these children is probably dependent on a series of environmental triggers as proposed by the “fertile field” hypothesis, and the unselected population in the ABIS cohort is uniquely suited to study these associations. This low-risk cohort is an often-overlooked population, as they are not usually included in screening programs or prevention trials, and they appear to have a different sensitivity to childhood environmental exposures, like infections, than high-risk children. More research is needed to confirm these associations, but the results imply that screening programs to detect early stages of type 1 diabetes could benefit from a general population design as opposed to screening only first-degree relatives of type 1 diabetes individuals. Individuals without risk-associated HLA alleles might even respond better to certain preventative therapies than high-risk individuals due to their decreased susceptibility to autoimmunity.

The subgroup analysis of high and increased risk individuals yields few significant associations between infections and development of type 1 diabetes. The reference group reported more episodes of the common cold than the type 1 diabetes group, in favor of the hygiene hypothesis (24) and contrary to results from the TEDDY study linking respiratory tract infections in early childhood to development of autoimmunity in genetically at-risk children (17). As the common cold is one of the most prevalent infections in early childhood, this could represent a decreased viral load negatively affecting the self-tolerance of the developing immune system (35). The TEDDY study has also reported bidirectional associations between gastrointestinal infections in early childhood and islet autoimmunity, where timing of the infection before or after 1 year of age either increased or decreased the risk of autoimmunity up to 10 years (36). As detection of islet autoantibodies was not included in this study, a similar analysis could not be performed for this cohort. It is possible that the timing of the questionnaires was too infrequent to detect significant associations in this group.

Despite the theory that early childhood infections could have a greater impact on the risk of developing type 1 diabetes before puberty, the results were similar for both onset before and after puberty. After adjusting for multiple testing, there were no differences in reported number or type of infections or antibiotics compared to the reference group.

Males with frequent episodes of gastroenteritis in early childhood had an increased risk of type 1 diabetes like the overall cohort, while females with type 1 diabetes had no significant associations with gastroenteritis or any other infections in any age group. Epidemiological studies have found more robust immune responses, decreased prevalence of certain persistent viruses and decreased viral loads in females compared to males (37). These differences are partly attributed to X-linked immune-associated genes (37).

### Strengths and limitations

ABIS is a prospective birth cohort including the general population. The aim was to study immune-mediated diseases, not just type 1 diabetes, eliminating the risk of selection bias. Participating families were not informed of any prior hypotheses or specific areas of study. The aim and design of this study will inherently result in multiple testing and risk of false discovery. An attempt to mitigate this was made by including possible confounders in the logistic regression analyses and performing a Benjamini & Hochberg procedure to determine statistical significance.

Self-reported data could suffer from recall bias, with an increased risk of under-reporting in cases of acute illness such as infections compared to chronic conditions (38). The relatively long intervals of approximately two years between questionnaires could amplify this risk. In particular, minor illnesses might be under-reported. It is possible that family constellation, age and number of siblings, cultural differences and coexisting chronic diseases might affect a carer’s likelihood to recall and report infections. However, the participation rate between families of children with type 1 diabetes and the reference group was similar for all age groups, and excluding infections registered after a diagnosis of type 1 diabetes eliminates the effect of systematic under-reporting for either group (or recall bias). In addition, the reported occurrence of minor viral infections like the common cold was consistently over 90% for all age groups, suggesting that the effect of under-reporting was likely small.

The self-reported data in the ABIS questionnaires was intended to cover all infectious episodes, including minor self-limiting infections, allowing for analysis of associations between childhood infections and type 1 diabetes that would not be possible using only medical records. Meanwhile, the NPR includes data from hospital admissions and specialized outpatient visits, likely underestimating the occurrence of minor infections but includes almost all major infections and complements the data from the ABIS questionnaires. Excluding infections registered after a diagnosis of type 1 diabetes mitigates the risk of reverse causation.

Methodologically, it would have been preferable to perform a Cox regression analysis of the entire period from 0-5 years to investigate the effect of a total infectious load during early childhood, however the assumption of proportional hazards over time was not met in this study. The lack of data on exact age, duration and associated symptoms of each infection reported in the questionnaires, in addition to unevenness in the extent of missing data between questionnaires, prohibited the use of a Cox regression analysis with time-dependent variables or other sensitivity analyses. For these reasons, a separate logistic regression analysis of each time period was performed instead. This approach yielded more comparisons, which might result in lack of significance after correcting for FDR using the Benjamini-Hochberg method. However, the small sample sizes, especially in subgroup analyses, warrants cautious interpretation and the risk of a type I error was deemed larger than the risk of a type II error.

The outcome of type 1 diabetes was determined by a clinical diagnosis requiring both symptoms of hyperglycemia and laboratory confirmation. This is considered a late stage of the disease, preceded by months or years of islet autoimmunity. It was not possible to distinguish whether early childhood infections had an effect on onset of islet autoimmunity or progression to overt type 1 diabetes in this study, but the study period of 0-5 years coincides with the peak age of seroconversion (39) and only a small proportion of cases progressed to manifest type 1 diabetes during the study period.

The greatest strength of using a general population cohort is the inclusion of individuals not followed in other high-risk cohorts, allowing for greater generalizability and the possibility to study the often-neglected group of type 1 diabetes cases without risk-associated HLA alleles. While this group is small, the diagnosis of type 1 diabetes in these individuals is just as life-altering as for anyone else, and they are rarely included in observational or prevention studies.

## Conclusion

Type and frequency of reported infections in the ABIS questionnaires was similar between both cases with type 1 diabetes and the reference group for all ages 0-5 years. Based on our results, infections and antibiotic use in early childhood do not seem to have a major impact in developing manifest type 1 diabetes in the general population, but individuals without risk-associated HLA alleles could be more sensitive to the effects of childhood infections. The association to frequent episodes of gastroenteritis in early childhood needs to be reproduced in other studies to be confirmed.

## Data Availability

The raw data supporting the conclusions of this article will be made available by the authors, without undue reservation.
