# Supplementary material for: Infections and antibiotic use in early childhood have limited importance in developing manifest type 1 diabetes – The ABIS cohort study
Source: Front Endocrinol (Lausanne). 2025 Feb 21;16:1529447. doi: 10.3389/fendo.2025.1529447 (PMC11885132; doi:10.3389/fendo.2025.1529447)
Supplement: Supplementary file 1 [file Table1.docx]

**Infections and antibiotic use in early childhood have limited importance in**

**developing manifest type 1 diabetes –**

**The ABIS cohort study**

**Supplemental table 1: Infections during 0-5 years of life**

|  | Type 1 diabetes  n (%) | Reference group  n (%) | OR  (95 % CI) | p-value | Adj. OR^a^  (95 % CI) | Adj.  p-value^a^ |
| --- | --- | --- | --- | --- | --- | --- |
| Total | 168 | 16260 |  |  |  |  |
| 1-12 months |  |  |  |  |  |  |
| Common cold |  |  |  |  |  |  |
| 1-2 times | 32 (31.4) | 3515 (33.9) | 1.58 (0.22-11.66) | 0.65 | 1.55 (0.21-11.46) | 0.67 |
| 3-5 times | 54 (52.9) | 4988 (48.1) | 1.88 (0.26-13.70) | 0.53 | 1.75 (0.24-12.79) | 0.58 |
| 6 or more times | 15 (14.7) | 1702 (16.4) | 1.53 (0.20-11.68) | 0.68 | 1.41 (0.18-10.83) | 0.74 |
| Otitis media | 27 (27.6) | 2581 (26.4) | 1.06 (0.68-1.66) | 0.79 | 1.07 (0.68-1.68) | 0.77 |
| Pneumonia | 4 (4.3) | 611 (6.6) | 0.63 (0.23-1.73) | 0.37 | 0.62 (0.23-1.70) | 0.35 |
| Influenza |  |  |  |  |  |  |
| 1-2 times | 12 (13.5) | 1122 (12.7) | 1.08 (0.58-1.99) | 0.81 | 1.13 (0.61-2.10) | 0.69 |
| 3-5 times | 1 (1.1) | 58 (0.7) | 1.74 (0.24-12.69) | 0.59 | 1.16 (0.15-9.20) | 0.89 |
| Gastroenteritis | 35 (34.7) | 3011 (29.4) | 1.28 (0.85-1.93) | 0.25 | 1.30 (0.85-1.96) | 0.22 |
| 1-2 times | 29 (32.2) | 2759 (30.0) | 1.13 (0.73-1.77) | 0.58 | 1.15 (0.73-1.80) | 0.56 |
| 3-5 times | 2 (2.2) | 72 (0.8) | 3.00 (0.72-12.50) | 0.13 | 3.86 (0.92-16.21) | 0.07 |
| Antibiotics | 44 (47.3) | 3788 (39.7) | 1.36 (0.90-2.05) | 0.14 | 1.39 (0.92-2.11) | 0.12 |
| Bacterial infection | 46 (44.7) | 4030 (38.4) | 1.29 (0.88-1.91) | 0.2 | 1.32 (0.89-1.96) | 0.17 |
| Viral infection | 106 (99.1) | 10654 (98.0) | 2.19 (0.30-15.75) | 0.44 | 2.08 (0.29-15.02) | 0.47 |
| 1 to 3 years |  |  |  |  |  |  |
| Common cold |  |  |  |  |  |  |
| 1-2 times | 16 (18.4) | 968 (11.2) | 1.72 (0.94-3.12) | 0.08 | 1.63 (0.87-3.04) | 0.13 |
| 3-5 times | 37 (42.5) | 4103 (47.6) | 0.94 (0.59-1.50) | 0.78 | 0.96 (0.60-1.56) | 0.88 |
| 6 or more times | 34 (39.1) | 3530 (41.0) | 0.58 (0.32-1.06) | 0.07 | 0.61 (0.33-1.15) | 0.12 |
| Tonsillitis |  |  |  |  |  |  |
| 1-2 times | 11 (14.1) | 1142 (14.7) | 0.93 (0.49-1.77) | 0.83 | 1.02 (0.53-1.95) | 0.96 |
| Otitis media |  |  |  |  |  |  |
| 1-2 times | 36 (41.4) | 3060 (37.9) | 1.09 (0.70-1.71) | 0.71 | 1.11 (0.70-1.77) | 0.65 |
| 3-5 times | 8 (9.2) | 839 (10.4) | 0.88 (0.41-1.89) | 0.75 | 0.87 (0.40-1.89) | 0.73 |
| 6 or more times | 1 (1.1) | 285 (3.5) | 0.33 (0.05-2.37) | 0.27 | 0.29 (0.04-2.14) | 0.22 |
| Pneumonia |  |  |  |  |  |  |
| 1-2 times | 10 (12.5) | 556 (7.1) | 1.86 (0.96-3.63) | 0.07 | 1.64 (0.81-3.34) | 0.17 |
| Influenza |  |  |  |  |  |  |
| 1-2 times | 26 (33.3) | 2727 (35.2) | 0.90 (0.56-1.45) | 0.67 | 0.86 (0.53-1.41) | 0.55 |
| 3-5 times | 3 (3.8) | 342 (4.4) | 0.83 (0.26-2.67) | 0.75 | 0.89 (0.27-2.90) | 0.85 |
| Gastroenteritis | 63 (75.0) | 6062 (73.4) | 1.09 (0.67-1.78) | 0.74 | 1.12 (0.67-1.87) | 0.67 |
| 1-2 times | 41 (49.4) | 5028 (60.9) | 0.86 (0.51-1.45) | 0.57 | 0.91 (0.53-1.56) | 0.72 |
| 3-5 times | 17 (20.5) | 956 (11.6) | 1.87 (0.98-3.56) | 0.06 | 1.77 (0.90-3.51) | 0.1 |
| 6 or more times | 4 (4.8) | 65 (0.8) | 6.46 (2.16-19.36) | **<0.001** | 8.21 (2.70-25.01) | **<0.001** |
| Antibiotics | 60 (67.4) | 5783 (66.6) | 1.04 (0.67-1.62) | 0.87 | 1.05 (0.66-1.66) | 0.83 |
| 1-2 times | 40 (49.4) | 3381 (42.2) | 1.31 (0.81-2.12) | 0.27 | 1.39 (0.85-2.28) | 0.19 |
| 3-5 times | 9 (11.1) | 1108 (13.8) | 0.90 (0.42-1.90) | 0.78 | 0.74 (0.33-1.66) | 0.47 |
| 6 or more times | 3 (3.7) | 317 (4.0) | 1.05 (0.32-3.45) | 0.94 | 0.95 (0.28-3.20) | 0.95 |
| Bacterial infection | 60 (69.8) | 5814 (69.5) | 1.01 (0.64-1.61) | 0.96 | 1.04 (0.65-1.68) | 0.87 |
| Viral infection | 76 (87.4) | 7170 (85.3) | 1.19 (0.63-2.25) | 0.59 | 1.12 (0.59-2.13) | 0.73 |
| 3 to 5 years |  |  |  |  |  |  |
| Common cold |  |  |  |  |  |  |
| 1-2 times | 7 (10.1) | 511 (7.1) | 1.91 (0.82-4.45) | 0.13 | 1.88 (0.80-4.41) | 0.15 |
| 3-5 times | 37 (53.6) | 3218 (44.5) | 1.61 (0.96-2.67) | 0.07 | 1.44 (0.85-2.44) | 0.17 |
| 6 or more times | 25 (36.2) | 3491 (48.3) | 0.52 (0.23-1.22) | 0.13 | 0.53 (0.23-1.26) | 0.15 |
| Tonsillitis |  |  |  |  |  |  |
| 1-2 times | 11 (17.5) | 1378 (19.9) | 0.82 (0.43-1.58) | 0.55 | 0.81 (0.41-1.62) | 0.56 |
| Otitis media |  |  |  |  |  |  |
| 1-2 times | 16 (25.0) | 2533 (36.3) | 0.52 (0.29-0.93) | 0.03* | 0.49 (0.27-0.90) | 0.02 |
| 3-5 times | 2 (3.1) | 711 (10.2) | 0.23 (0.06-0.96) | 0.04* | 0.21 (0.05-0.89) | 0.03 |
| 6 or more times | 3 (4.7) | 189 (2.7) | 1.31 (0.40-4.27) | 0.65 | 1.18 (0.35-3.93) | 0.79 |
| Pneumonia |  |  |  |  |  |  |
| 1-2 times | 5 (7.9) | 360 (5.2) | 1.59 (0.63-4.00) | 0.32 | 1.40 (0.55-3.58) | 0.49 |
| Influenza |  |  |  |  |  |  |
| 1-2 times | 24 (36.4) | 2720 (39.7) | 0.97 (0.57-1.64) | 0.9 | 0.98 (0.57-1.69) | 0.93 |
| 3-5 times | 8 (12.1) | 459 (6.7) | 1.91 (0.88-4.16) | 0.1 | 2.04 (0.92-4.53) | 0.08 |
| 6 or more times | 1 (1.5) | 50 (0.7) | 2.19 (0.29-16.36) | 0.44 | 2.72 (0.36-20.50) | 0.33 |
| Gastroenteritis | 65 (92.9) | 6352 (88.2) | 1.74 (0.70-4.34) | 0.23 | 2.16 (0.78-5.99) | 0.14 |
| 1-2 times | 40 (58.0) | 4390 (61.8) | 1.77 (0.63-4.97) | 0.28 | 2.24 (0.69-7.29) | 0.18 |
| 3-5 times | 24 (34.8) | 1768 (24.9) | 2.64 (0.91-7.64) | 0.07 | 3.51 (1.05-11.75) | 0.04 |
| 6 or more times | 1 (1.4) | 163 (2.3) | 1.19 (0.13-10.75) | 0.88 | 1.64 (0.17-16.02) | 0.67 |
| Antibiotics | 48 (68.6) | 5197 (71.1) | 0.89 (0.54-1.48) | 0.65 | 0.92 (0.55-1.56) | 0.77 |
| 1-2 times | 33 (53.2) | 2989 (43.4) | 1.34 (0.78-2.31) | 0.29 | 1.38 (0.79-2.41) | 0.26 |
| 3-5 times | 5 (8.1) | 948 (13.8) | 0.64 (0.24-1.70) | 0.37 | 0.68 (0.25-1.81) | 0.44 |
| 6 or more times | 2 (3.2) | 268 (3.9) | 0.91 (0.21-3.88) | 0.9 | 0.96 (0.22-4.15) | 0.95 |
| Bacterial infection | 46 (68.7) | 5240 (73.0) | 0.81 (0.48-1.36) | 0.42 | 0.77 (0.45-1.31) | 0.33 |
| Viral infection | 67 (95.7) | 6582 (91.4) | 2.10 (0.66-6.70) | 0.21 | 3.01 (0.73-12.37) | 0.13 |
| National Patient Register |  |  |  |  |  |  |
| Respiratory Tract Infection | 24 (14.3) | 2481 (15.3) | 0.93 (0.60-1.43) | 0.73 | 0.61 (0.30-1.23) | 0.16 |
| 1-2 times | 23 (13.7) | 2378 (14.6) | 0.93 (0.60-1.44) | 0.73 | 0.64 (0.32-1.29) | 0.21 |
| 3 or more times | 1 (0.6) | 103 (0.6) | 0.93 (0.13-6.70) | 0.94 | 0.83 (0.11-6.08) | 0.86 |
| Gastroenteritis | 4 (2.4) | 219 (1.3) | 1.79 (0.66-4.86) | 0.26 | 1.54 (0.36-6.56) | 0.56 |
| Urinary Tract Infection | 4 (2.4) | 229 (1.4) | 1.71 (0.63-4.64) | 0.3 | 2.14 (0.52-8.87) | 0.29 |
| Unspecified infection | 12 (7.1) | 929 (5.7) | 1.27 (0.70-2.29) | 0.43 | 1.85 (0.91-3.75) | 0.09 |
| Total infection | 33 (19.6) | 3398 (20.9) | 0.93 (0.63-1.36) | 0.69 | 0.79 (0.46-1.37) | 0.41 |
| 1-2 times | 27 (16.1) | 3126 (19.2) | 0.82 (0.54-1.25) | 0.36 | 0.74 (0.42-1.33) | 0.32 |
| 3 or more times | 6 (3.6) | 272 (1.7) | 2.10 (0.92-4.80) | 0.08 | 1.48 (0.35-6.20) | 0.6 |
| Bacterial infection | 13 (7.7) | 1442 (8.9) | 0.86 (0.49-1.52) | 0.61 | 0.80 (0.35-1.86) | 0.61 |
| 1-2 times | 12 (7.1) | 1414 (8.7) | 0.81 (0.45-1.46) | 0.49 | 0.82 (0.35-1.89) | 0.64 |
| 3 or more times | 1 (0.6) | 28 (0.2) | 3.41 (0.46-25.25) | 0.23 | 3.75 (0.51-27.75) | 0.20 |
| Viral infection | 30 (17.9) | 2421 (14.9) | 1.24 (0.84-1.85) | 0.28 | 1.09 (0.62-1.92) | 0.77 |
| 1-2 times | 29 (17.3) | 2331 (14.3) | 1.25 (0.83-1.87) | 0.28 | 1.13 (0.64-1.99) | 0.68 |
| 3 or more times | 1 (0.6) | 90 (0.6) | 1.11 (0.15-8.05) | 0.92 | 1.08 (0.15-7.91) | 0.94 |

*^a^Adjusted OR and adjusted p-values are calculated in a logistic regression analysis with sex, family history of type 1 diabetes and maternal education at birth. Bolded p-values are considered statistically significant after Benjamini & Hochberg correction for multiple comparisons.*

**Supplemental table 2: High or increased genetic risk of type 1 diabetes**

|  | Type 1 diabetes  n (%) | Reference group  n (%) | OR  (95 % CI) | p-value | Adj. OR^a^  (95 % CI) | Adj.  p-value^a^ |
| --- | --- | --- | --- | --- | --- | --- |
| Total | 91 (86.7) | 1470 (38.3) |  |  |  |  |
| 1-12 months |  |  |  |  |  |  |
| Common cold |  |  |  |  |  |  |
| 1-2 times | 19 (29.2) | 376 (31.1) | 1.16 (0.15-9.07) | 0.89 | 1.12 (0.14-8.81) | 0.92 |
| 3-5 times | 35 (53.8) | 602 (49.8) | 1.34 (0.18-10.19) | 0.78 | 1.21 (0.16-9.27) | 0.86 |
| 6 or more times | 10 (15.4) | 207 (17.1) | 1.11 (0.14-9.08) | 0.92 | 0.87 (0.10-7.18) | 0.89 |
| Otitis media | 16 (25.4) | 313 (27.4) | 0.90 (0.50-1.61) | 0.73 | 0.97 (0.53-1.76) | 0.92 |
| Pneumonia | 2 (3.3) | 69 (6.4) | 0.50 (0.12-2.09) | 0.34 | 0.54 (0.13-2.31) | 0.41 |
| Influenza |  |  |  |  |  |  |
| 1-2 times | 9 (15.8) | 123 (11.7) | 1.43 (0.68-2.99) | 0.34 | 1.62 (0.76-3.46) | 0.21 |
| 3-5 times | 1 (1.8) | 6 (0.6) | 3.26 (0.38-27.59) | 0.28 | 2.15 (0.20-23.08) | 0.53 |
| Gastroenteritis | 20 (31.7) | 375 (31.6) | 1.01 (0.58-1.74) | 0.98 | 1.06 (0.61-1.86) | 0.83 |
| 1-2 times | 18 (31.6) | 344 (31.8) | 0.98 (0.55-1.73) | 0.93 | 1.05 (0.58-1.90) | 0.86 |
| 3-5 times | 0 (0.0) | 12 (1.1) | na | 0.99 | na | 0.99 |
| Antibiotics | 27 (44.3) | 444 (40.0) | 1.19 (0.71-2.01) | 0.51 | 1.30 (0.76-2.24) | 0.34 |
| Bacterial infection | 27 (41.5) | 477 (39.2) | 1.10 (0.66-1.83) | 0.71 | 1.21 (0.71-2.04) | 0.48 |
| Viral infection | 66 (98.5) | 1233 (97.8) | 1.50 (0.20-11.19) | 0.69 | 1.38 (0.18-10.40) | 0.75 |
| 1 to 3 years |  |  |  |  |  |  |
| Common cold |  |  |  |  |  |  |
| 1-2 times | 28 (45.9) | 510 (46.0) | 0.41 (0.21-0.81) | **0.01** | 0.39 (0.19-0.80) | **0.01** |
| 3-5 times | 19 (31.1) | 494 (44.5) | 0.29 (0.14-0.59) | **<0.001** | 0.27 (0.13-0.58) | **<0.001** |
| Tonsillitis |  |  |  |  |  |  |
| 1-2 times | 9 (16.4) | 162 (16.2) | 0.99 (0.47-2.06) | 0.97 | 1.14 (0.54-2.43) | 0.73 |
| Otitis media |  |  |  |  |  |  |
| 1-2 times | 27 (43.5) | 376 (36.3) | 1.18 (0.69-2.01) | 0.55 | 1.24 (0.71-2.17) | 0.45 |
| 3-5 times | 3 (4.8) | 112 (10.8) | 0.44 (0.13-1.46) | 0.18 | 0.38 (0.11-1.32) | 0.13 |
| 6 or more times | 1 (1.6) | 40 (3.9) | 0.41 (0.06-3.09) | 0.39 | 0.53 (0.07-4.00) | 0.53 |
| Pneumonia |  |  |  |  |  |  |
| 1-2 times | 3 (5.4) | 73 (7.3) | 0.72 (0.22-2.36) | 0.59 | 0.83 (0.25-2.78) | 0.77 |
| Influenza |  |  |  |  |  |  |
| 1-2 times | 18 (33.3) | 338 (33.8) | 0.91 (0.51-1.64) | 0.76 | 0.87 (0.47-1.60) | 0.65 |
| 3-5 times | 1 (1.9) | 56 (5.6) | 0.31 (0.04-2.28) | 0.25 | 0.31 (0.04-2.33) | 0.25 |
| Gastroenteritis | 44 (73.3) | 802 (75.9) | 0.87 (0.49-1.58) | 0.66 | 0.88 (0.47-1.65) | 0.69 |
| 1-2 times | 32 (54.2) | 653 (61.8) | 0.79 (0.43-1.47) | 0.46 | 0.80 (0.42-1.54) | 0.51 |
| 3-5 times | 9 (15.3) | 135 (12.8) | 1.08 (0.46-2.50) | 0.87 | 1.11 (0.45-2.75) | 0.82 |
| 6 or more times | 2 (3.4) | 11 (1.0) | 2.93 (0.60-14.36) | 0.19 | 4.16 (0.82-21.13) | 0.09 |
| Antibiotics | 39 (61.9) | 754 (67.6) | 0.78 (0.46-1.32) | 0.35 | 0.83 (0.48-1.45) | 0.51 |
| 1-2 times | 32 (50.8) | 502 (45.9) | 0.97 (0.57-1.66) | 0.92 | 1.06 (0.61-1.87) | 0.83 |
| 3-5 times | 4 (6.3) | 159 (14.5) | 0.38 (0.13-1.12) | 0.08 | 0.37 (0.12-1.12) | 0.08 |
| 6 or more times | 1 (1.6) | 36 (3.3) | 0.42 (0.06-3.22) | 0.41 | 0.43 (0.05-3.49) | 0.43 |
| Bacterial infection | 38 (63.3) | 753 (70.3) | 0.73 (0.43-1.25) | 0.25 | 0.82 (0.46-1.46) | 0.5 |
| Viral infection | 53 (86.9) | 935 (87.0) | 0.99 (0.46-2.13) | 0.98 | 0.85 (0.39-1.87) | 0.69 |
| 3 to 5 years |  |  |  |  |  |  |
| Common cold |  |  |  |  |  |  |
| 1-2 times | 29 (58.0) | 393 41.9) | 0.78 (0.31-1.94) | 0.59 | 0.60 (0.23-1.54) | 0.28 |
| 3-5 times | 15 (30.0) | 482 (51.4) | 0.33 (0.12-0.87) | 0.03 | 0.25 (0.09-0.71) | 0.009 |
| Tonsillitis |  |  |  |  |  |  |
| 1-2 times | 9 (20.5) | 174 (19.2) | 1.04 (0.49-2.21) | 0.92 | 0.92 (0.40-2.11) | 0.85 |
| Otitis media |  |  |  |  |  |  |
| 1-2 times | 11 (24.4) | 321 (34.9) | 0.54 (0.27-1.09) | 0.09 | 0.52 (0.24-1.10) | 0.09 |
| 3-5 times | 1 (2.2) | 80 (8.7) | 0.20 (0.03-1.46) | 0.11 | 0.14 (0.02-1.14) | 0.07 |
| 6 or more times | 2 (4.4) | 30 (3.3) | 1.05 (0.24-4.60) | 0.95 | 1.03 (0.21-4.97) | 0.97 |
| Pneumonia |  |  |  |  |  |  |
| 1-2 times | 1 (2.3) | 52 (5.7) | 0.39 (0.05-2.90) | 0.36 | 0.23 (0.03-1.88) | 0.17 |
| Influenza |  |  |  |  |  |  |
| 1-2 times | 16 (34.8) | 332 (37.1) | 0.96 (0.51-1.83) | 0.91 | 0.86 (0.43-1.70) | 0.66 |
| 3-5 times | 4 (8.7) | 56 (6.3) | 1.43 (0.48-4.25) | 0.52 | 1.38 (0.45-4.26) | 0.57 |
| Gastroenteritis | 45 (90.0) | 830 (88.5) | 1.17 (0.46-3.01) | 0.74 | 1.56 (0.53-4.61) | 0.42 |
| 1-2 times | 33 (67.3) | 573 (62.1) | 1.40 (0.48-4.03) | 0.54 | 1.74 (0.51-5.80) | 0.38 |
| 3-5 times | 11 (22.4) | 236 (25.6) | 1.13 (0.35-3.64) | 0.84 | 1.52 (0.40-5.77) | 0.54 |
| 6 or more times | 1 (2.0) | 17 (1.8) | 1.43 (0.15-13.55) | 0.76 | 1.71 (0.15-18.96) | 0.66 |
| Antibiotics | 33 (66.0) | 676 (71.1) | 0.79 (0.43-1.44) | 0.44 | 0.84 (0.45-1.59) | 0.6 |
| 1-2 times | 21 (42.9) | 455 (49.1) | 0.78 (0.41-1.49) | 0.46 | 0.81 (0.41-1.59) | 0.53 |
| 3-5 times | 9 (18.4) | 136 (14.7) | 1.12 (0.49-2.56) | 0.79 | 1.08 (0.45-2.60) | 0.86 |
| 6 or more times | 1 (2.0) | 30 (3.2) | 0.57 (0.07-4.38) | 0.59 | 0.70 (0.09-5.55) | 0.74 |
| Bacterial infection | 32 (68.1) | 675 (72.0) | 0.83 (0.44-1.55) | 0.56 | 0.80 (0.41-1.55) | 0.51 |
| Viral infection | 47 (94.0) | 857 (91.8) | 1.41 (0.43-4.63) | 0.57 | 2.21 (0.50-9.75) | 0.3 |
| National Patient Register |  |  |  |  |  |  |
| Respiratory Tract Infection | 10 (11.0) | 238 (16.2) | 0.64 (0.33-1.25) | 0.19 | 0.15 (0.03-0.62) | 0.009 |
| 1-2 times | 9 (9.9) | 227 (15.4) | 0.60 (0.30-1.22) | 0.16 | 0.16 (0.04-0.67) | 0.01 |
| 3 or more times | 1 (1.1) | 11 (0.7) | 1.38 (0.18-10.84) | 0.76 | 1.06 (0.12-9.21) | 0.96 |
| Gastroenteritis | 4 (4.4) | 23 (1.6) | 2.89 (0.98-8.55) | 0.06 | 1.75 (0.39-7.90) | 0.47 |
| Urinary Tract Infection | 2 (2.2) | 23 (1.6) | 1.41 (0.33-6.09) | 0.64 | 1.38 (0.18-10.63) | 0.76 |
| Unspecified infection | 4 (4.4) | 92 (6.3) | 0.69 (0.25-1.92) | 0.48 | 0.94 (0.32-2.76) | 0.91 |
| Total infection | 16 (17.6) | 331 (22.5) | 0.73 (0.42-1.28) | 0.27 | 0.39 (0.17-0.89) | 0.03 |
| 1-2 times | 13 (14.3) | 304 (20.7) | 0.65 (0.36-1.19) | 0.16 | 0.36 (0.15-0.87) | 0.02 |
| 3 or more times | 3 (3.3) | 27 (1.8) | 1.69 (0.50-5.69) | 0.4 | 0.71 (0.09-5.86) | 0.75 |
| Bacterial infection | 7 (7.7) | 133 (9.0) | 0.84 (0.38-1.85) | 0.66 | 0.46 (0.14-1.55) | 0.21 |
| 1-2 times | 7 (7.7) | 132 (9.0) | 0.84 (0.38-1.86) | 0.68 | 0.47 (0.14-1.57) | 0.22 |
| Viral infection | 14 (15.4) | 235 (16.0) | 0.96 (0.53-1.72) | 0.88 | 0.51 (0.21-1.22) | 0.13 |
| 1-2 times | 14 (15.4) | 225 (15.3) | 1.00 (0.56-1.80) | 0.99 | 0.54 (0.22-1.30) | 0.17 |

*^a^Adjusted OR and adjusted p-values are calculated in a logistic regression analysis with sex, family history of type 1 diabetes and maternal education at birth. Bolded p-values are considered statistically significant after Benjamini & Hochberg correction for multiple comparisons.*

**Supplemental table 3: Neutral or decreased genetic risk of type 1 diabetes**

|  | Type 1 diabetes  n (%) | Reference group  n (%) | OR  (95 % CI) | p-value | Adj. OR^a^  (95 % CI) | Adj.  p-value^a^ |
| --- | --- | --- | --- | --- | --- | --- |
| Total | 14 (13.3) | 2372 (61.7) |  |  |  |  |
| 1-12 months |  |  |  |  |  |  |
| Common cold |  |  |  |  |  |  |
| 1-2 times | 3 (23.1) | 629 (32.5) | 0.77 (0.13-4.68) | 0.99 | 0.83 (0.13-5.12) | 0.84 |
| 3-5 times | 8 (61.5) | 956 (49.4) | 1.76 (0.46-6.64) | 0.41 | 1.75 (0.46-6.76) | 0.41 |
| 6 or more times | 2 (15.4) | 326 (16.8) | 1.29 (0.21-7.74) | 0.78 | 1.21 (0.20-7.45) | 0.84 |
| Otitis media | 4 (33.3) | 485 (26.9) | 1.36 (0.41-4.53) | 0.62 | 1.38 (0.41-4.70) | 0.61 |
| Pneumonia | 2 (18.2) | 109 (6.3) | 3.28 (0.70-15.36) | 0.13 | 4.20 (0.86-20.57) | 0.08 |
| Influenza |  |  |  |  |  |  |
| 1-2 times | 1 (7.7) | 220 (13.2) | 0.55 (0.07-4.21) | 0.56 | 0.63 (0.08-4.97) | 0.66 |
| 3-5 times | 0 (0.0) | 8 (0.5) | na | 0.99 | na | 0.99 |
| Gastroenteritis | 4 (30.8) | 624 (32.7) | 0.92 (0.28-2.98) | 0.88 | 0.89 (0.27-2.97) | 0.85 |
| 1-2 times | 3 (23.1) | 567 (32.8) | 0.67 (0.18-2.50) | 0.56 | 0.61 (0.16-2.33) | 0.46 |
| 3-5 times | 1 (7.7) | 15 (0.9) | 8.49 (1.01-71.28) | 0.05 | 13.66 (1.54-120.84) | 0.02 |
| Antibiotics | 9 (69.2) | 699 (39.3) | 3.47 (1.07-11.32) | 0.04 | 3.21 (0.97-10.67) | 0.06 |
| Bacterial infection | 9 (69.2) | 744 (38.3) | 3.62 (1.11-11.81) | 0.03 | 3.66 (1.10-12.13) | 0.03 |
| Viral infection | 13 (100.0) | 1983 (98.5) | na | 0.99 | na | 0.99 |
| 1 to 3 years |  |  |  |  |  |  |
| Common cold |  |  |  |  |  |  |
| 1-2 times | 2 (20.0) | 192 (10.6) | 1.27 (0.26-6.36) | 0.77 | 1.42 (0.28-7.35) | 0.67 |
| 3-5 times | 2 (20.0) | 876 (48.5) | 0.22 (0.03-1.57) | 0.13 | 0.23 (0.03-1.65) | 0.14 |
| 6 or more times | 6 (60.0) | 733 (40.5) | 0.79 (0.16-3.92) | 0.77 | 0.70 (0.14-3.63) | 0.67 |
| Tonsillitis |  |  |  |  |  |  |
| 1-2 times | 0 (0.0) | 235 (14.4) | na | 0.99 | na | 0.99 |
| Otitis media |  |  |  |  |  |  |
| 1-2 times | 4 (40.0) | 622 (36.7) | 1.37 (0.34-5.48) | 0.66 | 1.50 (0.36-6.29) | 0.58 |
| 3-5 times | 2 (20.0) | 161 (9.5) | 2.64 (0.48-14.52) | 0.27 | 2.49 (0.42-14.81) | 0.32 |
| 6 or more times | 0 (0.0) | 65 (3.8) | na | 0.99 | na | 0.99 |
| Pneumonia |  |  |  |  |  |  |
| 1-2 times | 5 (55.6) | 88 (5.4) | 21.99 (5.80-83.32) | **<0.001** | 26.08 (6.29-108.17) | **<0.001** |
| Influenza |  |  |  |  |  |  |
| 1-2 times | 3 (33.3) | 583 (35.7) | 0.83 (0.21-3.33) | 0.79 | 1.07 (0.25-4.52) | 0.93 |
| 3-5 times | 0 (0.0) | 68 (4.2) | na | 0.99 | na | 0.99 |
| Gastroenteritis | 6 (60.0) | 1278 (73.6) | 0.54 (0.15-1.92) | 0.34 | 0.68 (0.18-2.60) | 0.57 |
| 1-2 times | 1 (10.0) | 1059 (61.0) | 0.11 (0.01-0.98) | 0.05 | 0.14 (0.02-1.29) | 0.08 |
| 3-5 times | 4 (40.0) | 202 (11.6) | 2.29 (0.57-9.24) | 0.25 | 2.47 (0.57-10.75) | 0.23 |
| 6 or more times | 1 (10.0) | 14 (0.8) | 8.25 (0.87-78.66) | 0.07 | 12.58 (1.17-134.75) | 0.04 |
| Antibiotics | 8 (80.0) | 1171 (63.8) | 2.27 (0.48-10.73) | 0.3 | 2.61 (0.53-13.01) | 0.24 |
| 1-2 times | 4 (40.0) | 805 (44.9) | 0.90 (0.22-3.61) | 0.88 | 0.96 (0.23-3.99) | 0.96 |
| 3-5 times | 1 (10.0) | 199 (11.1) | 0.91 (0.10-8.18) | 0.93 | 1.07 (0.11-10.02) | 0.95 |
| 6 or more times | 1 (10.0) | 63 (3.5) | 2.87 (0.32-26.10) | 0.35 | 1.47 (0.14-15.40) | 0.75 |
| Bacterial infection | 8 (80.0) | 1158 (65.9) | 2.07 (0.44-9.77) | 0.36 | 2.39 (0.48-11.92) | 0.29 |
| Viral infection | 7 (70.0) | 1510 (85.3) | 0.40 (0.10-1.57) | 0.19 | 0.47 (0.11-1.93) | 0.29 |
| 3 to 5 years |  |  |  |  |  |  |
| Common cold |  |  |  |  |  |  |
| 1-2 times | 0 (0.0) | 114 (7.6) | na | 0.99 | na | 0.99 |
| 3-5 times | 2 (40.0) | 695 (46.1) | 0.67 (0.11-3.99) | 0.66 | 0.55 (0.08-3.71) | 0.54 |
| 6 or more times | 3 (60.0) | 693 (46.0) | 1.51 (0.25-9.03) | 0.66 | 1.81 (0.27-12.13) | 0.54 |
| Tonsillitis |  |  |  |  |  |  |
| 1-2 times | 0 (0.0) | 303 (20.7) | na | 0.99 | na | 0.99 |
| Otitis media |  |  |  |  |  |  |
| 1-2 times | 2 (40.0) | 555 (38.0) | 0.91 (0.15-5.43) | 0.91 | 1.51 (0.21-10.67) | 0.68 |
| 3-5 times | 0 (0.0) | 118 (8.1) | na | 0.99 | na | 0.99 |
| 6 or more times | 0 (0.0) | 34 (2.3) | na | 0.99 | na | 0.99 |
| Pneumonia |  |  |  |  |  |  |
| 1-2 times | 2 (40.0) | 60 (4.1) | 15.39 (2.52-93.82) | **0.003** | 35.63 (4.10-309.96) | **0.001** |
| Influenza |  |  |  |  |  |  |
| 1-2 times | 1 (20.0) | 585 (40.9) | 0.65 (0.06-7.13) | 0.72 | 0.50 (0.04-6.33) | 0.59 |
| 3-5 times | 2 (40.0) | 82 (5.7) | 9.21 (1.28-66.23) | 0.03 | 14.68 (1.58-135.99) | 0.02 |
| Gastroenteritis | 5 (100.0) | 1329 (88.1) | na | 0.99 | na | 0.99 |
| 1-2 times | 1 (20.0) | 929 (62.3) | 0.10 (0.01-0.87) | 0.04 | 0.05 (0.004-0.73) | 0.03 |
| 3-5 times | 4 (80.0) | 359 (24.1) | 10.35 (1.15-92.92) | 0.04 | 19.05 (1.37-263.90) | 0.03 |
| 6 or more times | 0 (0.0) | 32 (2.1) | na | 0.99 | na | 0.99 |
| Antibiotics | 4 (80.0) | 1077 (70.4) | 1.68 (0.19-15.06) | 0.64 | 2.23 (0.22-22.32) | 0.5 |
| 1-2 times | 2 (50.0) | 743 (49.9) | 0.67 (0.09-4.79) | 0.69 | 0.82 (0.10-6.59) | 0.85 |
| 3-5 times | 0 (0.0) | 199 (13.4) | na | 0.99 | na | 0.99 |
| 6 or more times | 0 (0.0) | 48 (3.2) | na | 0.99 | na | 0.99 |
| Bacterial infection | 4 (80.0) | 1082 (72.1) | 1.55 (0.17-13.87) | 0.7 | 1.85 (0.19-18.34) | 0.6 |
| Viral infection | 5 (100.0) | 1371 (90.9) | na | 0.99 | na | 0.99 |
| National Patient Register |  |  |  |  |  |  |
| Respiratory Tract Infection | 5 (35.7) | 343 (14.5) | 3.29 (1.10-9.87) | **0.03** | 4.98 (1.31-18.95) | **0.02** |
| 1-2 times | 5 (35.7) | 334 (14.1) | 3.38 (1.12-10.13) | **0.03** | 5.14 (1.35-19.56) | **0.02** |
| Gastroenteritis | 0 (0.0) | 42 (1.8) | na | 0.99 | na | 0.99 |
| Urinary Tract Infection | 1 (7.1) | 26 (1.1) | 6.94 (0.88-55.02) | 0.07 | 9.39 (1.05-83.77) | **0.05** |
| Unspecified infection | 3 (21.4) | 135 (5.7) | 4.52 (1.25-16.39) | 0.02 | 7.51 (1.81-31.11) | **0.005** |
| Total infection | 6 (42.9) | 486 (20.5) | 2.91 (1.01-8.43) | 0.05 | 3.77 (1.06-13.47) | **0.04** |
| 1-2 times | 5 (35.7) | 455 (19.2) | 2.59 (0.84-7.96) | 0.1 | 3.10 (0.80-12.02) | 0.1 |
| 3 or more times | 1 (7.1) | 31 (1.3) | 7.61 (0.92-62.66) | 0.06 | 18.71 (1.95-179.55) | **0.01** |
| Bacterial infection | 1 (7.1) | 187 (7.9) | 0.90 (0.12-6.91) | 0.92 | 1.43 (0.17-11.70) | 0.74 |
| 1-2 times | 1 (7.1) | 182 (7.7) | 0.92 (0.12-7.10) | 0.94 | 1.44 (0.18-11.79) | 0.74 |
| 3 or more times | 0 (0.0) | 5 (0.2) | na | 0.99 | na | 0.99 |
| Viral infection | 6 (42.9) | 355 (15.0) | 4.26 (1.47-12.36) | **0.008** | 5.72 (1.59-20.57) | **0.008** |
| 1-2 times | 6 (42.9) | 342 (14.4) | 4.42 (1.53-12.83) | **0.006** | 5.91 (1.64-21.29) | **0.007** |

*^a^Adjusted OR and adjusted p-values are calculated in a logistic regression analysis with sex, family history of type 1 diabetes and maternal education at birth. Bolded p-values are considered statistically significant after Benjamini & Hochberg correction for multiple comparisons.*

**Supplemental table 4: Onset of type 1 diabetes before puberty**

|  | Type 1 diabetes  n (%) | Reference group  n (%) | OR  (95 % CI) | p-value | Adj. OR^a^  (95 % CI) | Adj.  p-value^a^ |
| --- | --- | --- | --- | --- | --- | --- |
| **Total** | 72 (42.9) | 16260 |  |  |  |  |
| **1-12 months** |  |  |  |  |  |  |
| Common cold |  |  |  |  |  |  |
| 1-2 times | 8 (20.0) | 3515 (33.9) | 0.78 (0.25-2.37) | 0.65 | 0.81 (0.26-2.50) | 0.71 |
| 3-5 times | 27 (67.5) | 4988 (48.1) | 2.38 (1.08-5.24) | 0.03 | 2.32 (1.05-5.13) | 0.04 |
| 6 or more times | 5 (12.5) | 1702 (16.4) | 1.29 (0.42-3.95) | 0.66 | 1.23 (0.40-3.80) | 0.72 |
| Otitis media | 11 (28.2) | 2581 (26.4) | 1.10 (0.55-2.21) | 0.8 | 1.07 (0.53-2.16) | 0.86 |
| Pneumonia | 3 (7.7) | 611 (6.6) | 1.19 (0.36-3.86) | 0.78 | 1.13 (0.34-3.71) | 0.84 |
| Influenza |  |  |  |  |  |  |
| 1-2 times | 6 (17.1) | 1122 (12.7) | 1.46 (0.60-3.54) | 0.4 | 1.50 (0.61-3.65) | 0.38 |
| 3-5 times | 1 (2.9) | 58 (0.7) | 4.71 (0.63-35.19) | 0.13 | 2.42 (0.28-21.13) | 0.43 |
| Gastroenteritis | 16 (39.0) | 3011 (29.4) | 1.54 (0.82-2.89) | 0.18 | 1.55 (0.82-2.91) | 0.18 |
| 1-2 times | 13 (36.1) | 2759 (30.0) | 1.36 (0.69-2.71) | 0.38 | 1.36 (0.68-2.71) | 0.39 |
| 3-5 times | 1 (2.8) | 72 (0.8) | 4.02 (0.54-30.22) | 0.18 | 5.39 (0.71-40.94) | 0.1 |
| Antibiotics | 14 (40.0) | 3788 (39.7) | 1.01 (0.51-1.99) | 0.98 | 0.99 (0.50-1.97) | 0.98 |
| Bacterial infection | 15 (36.6) | 4030 (38.4) | 0.93 (0.49-1.75) | 0.81 | 0.91 (0.48-1.73) | 0.77 |
| Viral infection | 42 (100.0) | 10654 (98.0) | na | 0.99 | na | 0.99 |
| **1 to 3 years** |  |  |  |  |  |  |
| Common cold |  |  |  |  |  |  |
| 1-2 times | 6 (15.4) | 968 (11.2) | 1.46 (0.56-3.77) | 0.43 | 1.45 (0.55-3.86) | 0.46 |
| 3-5 times | 18 (46.2) | 4103 (47.6) | 1.03 (0.52-2.05) | 0.93 | 1.15 (0.57-2.33) | 0.70 |
| 6 or more times | 15 (38.5) | 3530 (41.0) | 0.69 (0.27-1.77) | 0.44 | 0.69 (0.26-1.83) | 0.45 |
| Tonsillitis |  |  |  |  |  |  |
| 1-2 times | 3 (8.3) | 1142 (14.7) | 0.52 (0.16-1.69) | 0.27 | 0.57 (0.17-1.89) | 0.36 |
| Otitis media |  |  |  |  |  |  |
| 1-2 times | 17 (41.5) | 3060 (37.9) | 1.14 (0.59-2.19) | 0.7 | 1.24 (0.63-2.43) | 0.53 |
| 3-5 times | 4 (9.8) | 839 (10.4) | 0.98 (0.33-2.88) | 0.97 | 0.97 (0.32-2.92) | 0.96 |
| 6 or more times | 1 (2.4) | 285 (3.5) | 0.72 (0.10-5.39) | 0.75 | 0.65 (0.08-4.94) | 0.67 |
| Pneumonia |  |  |  |  |  |  |
| 1-2 times | 6 (15.8) | 556 (7.1) | 2.44 (1.02-5.87) | 0.05 | 1.93 (0.74-5.05) | 0.18 |
| Influenza |  |  |  |  |  |  |
| 1-2 times | 12 (34.3) | 2727 (35.2) | 0.89 (0.44-1.78) | 0.73 | 0.89 (0.44-1.81) | 0.74 |
| 3-5 times | 0 (0.0) | 342 (4.4) | na | 0.99 | na | 0.99 |
| Gastroenteritis | 26 (70.3) | 6062 (73.4) | 0.86 (0.42-1.74) | 0.67 | 0.84 (0.41-1.72) | 0.63 |
| 1-2 times | 14 (38.9) | 5028 (60.9) | 0.68 (0.32-1.45) | 0.32 | 0.57 (0.25-1.26) | 0.16 |
| 3-5 times | 9 (25.0) | 956 (11.6) | 1.89 (0.78-4.57) | 0.16 | 1.73 (0.68-4.39) | 0.25 |
| 6 or more times | 2 (5.6) | 65 (0.8) | 6.17 (1.34-28.39) | 0.02 | 8.43 (1.79-39.66) | 0.007 |
| Antibiotics | 25 (64.1) | 5783 (66.6) | 0.90 (0.47-1.73) | 0.74 | 0.89 (0.46-1.74) | 0.74 |
| 1-2 times | 16 (41.0) | 3890 (45.8) | 0.77 (0.38-1.52) | 0.44 | 0.81 (0.40-1.62) | 0.55 |
| 3-5 times | 5 (12.8) | 1162 (13.7) | 0.80 (0.30-2.17) | 0.66 | 0.57 (0.19-1.73) | 0.32 |
| 6 or more times | 1 (2.6) | 278 (3.3) | 0.67 (0.09-5.05) | 0.7 | 0.57 (0.07-4.40) | 0.59 |
| Bacterial infection | 25 (64.1) | 5814 (69.5) | 0.78 (0.41-1.51) | 0.47 | 0.79 (0.40-1.54) | 0.49 |
| Viral infection | 33 (86.8) | 7170 (85.3) | 1.14 (0.44-2.92) | 0.79 | 1.09 (0.42-2.84) | 0.86 |
| **3 to 5 years** |  |  |  |  |  |  |
| Common cold |  |  |  |  |  |  |
| 1-2 times | 1 (3.6) | 511 (7.1) | 0.62 (0.08-4.82) | 0.65 | 0.58 (0.07-4.56) | 0.61 |
| 3-5 times | 16 (57.1) | 3218 (44.5) | 1.58 (0.73-3.41) | 0.25 | 1.42 (0.65-3.12) | 0.38 |
| 6 or more times | 11 (39.3) | 3491 (48.3) | 1.61 (0.21-12.50) | 0.64 | 1.72 (0.22-13.48) | 0.61 |
| Tonsillitis |  |  |  |  |  |  |
| 1-2 times | 3 (10.7) | 1378 (19.9) | 0.48 (0.14-1.58) | 0.22 | 0.51 (0.15-1.71) | 0.28 |
| Otitis media |  |  |  |  |  |  |
| 1-2 times | 8 (28.6) | 2533 (36.3) | 0.59 (0.26-1.35) | 0.21 | 0.55 (0.23-1.32) | 0.18 |
| 3-5 times | 0 (0.0) | 711 (10.2) | na | 0.99 | na | 0.99 |
| 6 or more times | 1 (3.6) | 189 (2.7) | 0.99 (0.13-7.43) | 0.99 | 0.84 (0.11-6.45) | 0.87 |
| Pneumonia |  |  |  |  |  |  |
| 1-2 times | 1 (3.6) | 360 (5.2) | 0.70 (0.09-5.16) | 0.73 | 0.56 (0.07-4.21) | 0.57 |
| 3-5 times | 1 (3.6) | 19 (0.3) | 13.23 (1.71-102.48) | 0.01 | 18.57 (2.35-146.88) | 0.006 |
| Influenza |  |  |  |  |  |  |
| 1-2 times | 10 (35.7) | 2720 (39.7) | 0.95 (0.42-2.14) | 0.9 | 0.99 (0.43-2.27) | 0.98 |
| 3-5 times | 3 (10.7) | 459 (6.7) | 1.69 (0.48-5.90 | 0.41 | 1.73 (0.48-6.23) | 0.4 |
| 6 or more times | 1 (3.6) | 50 (0.7) | 5.17 (0.67-40.09) | 0.12 | 6.32 (0.80-50.16) | 0.08 |
| Gastroenteritis | 26 (92.9) | 6352 (88.2) | 1.24 (0.28-5.47) | 0.78 | 1.74 (0.42-7.42) | 0.45 |
| 1-2 times | 14 (50.0) | 4390 (61.8) | 1.24 (0.28-5.47) | 0.78 | 1.16 (0.26-5.21) | 0.84 |
| 3-5 times | 11 (39.3) | 1768 (24.9) | 2.42 (0.54-10.95) | 0.25 | 2.45 (0.54-11.16) | 0.25 |
| 6 or more times | 1 (3.6) | 163 (2.3) | 2.39 (0.22-26.48) | 0.48 | 2.61 (0.23-29.35) | 0.44 |
| Antibiotics | 20 (71.4) | 5197 (71.1) | 1.02 (0.45-2.32) | 0.97 | 1.01 (0.44-2.32) | 0.99 |
| 1-2 times | 12 (44.4) | 3387 (47.5) | 0.92 (0.39-2.18) | 0.85 | 0.84 (0.35-2.04) | 0.7 |
| 3-5 times | 5 (18.5) | 1121 (15.7) | 1.16 (0.39-3.46) | 0.8 | 1.10 (0.36-3.31) | 0.87 |
| 6 or more times | 1 (3.7) | 290 (4.1) | 0.89 (0.11-7.08) | 0.92 | 0.80 (0.10-6.48) | 0.84 |
| Bacterial infection | 19 (67.9) | 5240 (73.0) | 0.78 (0.35-1.73) | 0.54 | 0.75 (0.33-1.68) | 0.48 |
| Viral infection | 27 (96.4) | 6582 (91.4) | 2.54 (0.34-18.72) | 0.36 | 2.40 (0.32-17.78) | 0.39 |
| **National Patient Register** |  |  |  |  |  |  |
| Respiratory Tract Infection | 9 (12.5) | 2481 (15.3) | 0.79 (0.39-1.60) | 0.52 | 0.28 (0.07-1.17) | 0.08 |
| 1-2 times | 8 (11.1) | 2378 (14.6) | 0.74 (0.35-1.54) | 0.41 | 0.30 (0.07-1.24) | 0.1 |
| 3 or more times | 1 (1.4) | 103 (0.6) | 2.12 (0.29-15.46) | 0.46 | 1.27 (0.54-2.96) | 0.58 |
| Gastroenteritis | 0 (0.0) | 219 (1.3) | na | 0.99 | na | 0.99 |
| Urinary Tract Infection | 2 (2.8) | 229 (1.4) | 2.00 (0.49-8.21) | 0.34 | 2.48 (0.33-18.44) | 0.37 |
| Unspecified infection | 4 (5.6) | 929 (5.7) | 0.97 (0.35-2.67) | 0.96 | 0.86 (0.21-3.61) | 0.84 |
| Total infection | 11 (15.3) | 3398 (20.9) | 0.68 (0.36-1.30) | 0.24 | 0.29 (0.09-0.93) | 0.04 |
| 1-2 times | 8 (11.1) | 3126 (19.2) | 0.54 (0.26-1.13) | 0.1 | 0.20 (0.05-0.85) | 0.03 |
| 3 or more times | 3 (4.2) | 272 (1.7) | 2.33 (0.73-7.46) | 0.16 | 1.41 (0.19-10.65) | 0.74 |
| Bacterial infection | 5 (6.9) | 1442 (8.9) | 0.77 (0.31-1.91) | 0.57 | 0.58 (0.14-2.43) | 0.46 |
| 1-2 times | 5 (6.9) | 1414 (8.7) | 0.78 (0.32-1.94) | 0.6 | 0.59 (0.14-2.47) | 0.47 |
| Viral infection | 11 (15.3) | 2421 (14.9) | 1.03 (0.54-1.96) | 0.93 | 0.42 (0.13-1.39) | 0.42 |
| 1-2 times | 10 (13.9) | 2331 (14.3) | 0.97 (0.50-1.90) | 0.94 | 0.44 (0.13-1.44) | 0.17 |
| 3 or more times | 1 (1.4) | 90 (0.6) | 2.52 (0.35-18.38) | 0.36 | 2.48 (0.34-18.35) | 0.37 |

*^a^Adjusted OR and adjusted p-values are calculated in a logistic regression analysis with sex, family history of type 1 diabetes and maternal education at birth. Bolded p-values are considered statistically significant after Benjamini & Hochberg correction for multiple comparisons.*

**Supplemental table 5: Onset of type 1 diabetes after puberty**

|  | Type 1 diabetes  n (%) | Reference group  n (%) | OR  (95 % CI) | p-value | Adj. OR^a^  (95 % CI) | Adj.  p-value^a^ |
| --- | --- | --- | --- | --- | --- | --- |
| Total | 96 (57.1) | 16260 |  |  |  |  |
| 1-12 months |  |  |  |  |  |  |
| Common cold |  |  |  |  |  |  |
| 1-2 times | 24 (38.7) | 3515 (33.9) | 1.19 (0.16-8.83) | 0.87 | 1.15 (0.15-8.60) | 0.89 |
| 3-5 times | 27 (43.5) | 4988 (48.1) | 0.94 (0.13-6.97) | 0.95 | 0.85 (0.11-6.33) | 0.88 |
| 6 or more times | 10 (16.1) | 1702 (16.4) | 1.02 (0.13-8.03) | 0.98 | 0.93 (0.12-7.34) | 0.94 |
| Otitis media | 16 (27.1) | 2581 (26.4) | 1.04 (0.58-1.85) | 0.9 | 1.07 (0.60-1.91) | 0.82 |
| Pneumonia | 1 (1.8) | 611 (6.6) | 0.26 (0.04-1.91) | 0.19 | 0.26 (0.04-1.89) | 0.18 |
| Influenza |  |  |  |  |  |  |
| 1-2 times | 6 (11.1) | 1122 (12.7) | 0.85 (0.36-2.00) | 0.71 | 0.92 (0.39-2.16) | 0.84 |
| 3-5 times | 0 (0.0) | 58 (0.7) | na | 0.99 | na | 0.99 |
| Gastroenteritis | 19 (31.7) | 3011 (29.4) | 1.11 (0.65-1.92) | 0.7 | 1.14 (0.66-1.97) | 0.65 |
| 1-2 times | 16 (29.6) | 2759 (30.0) | 1.00 (0.55-1.80) | 0.99 | 1.02 (0.56-1.84) | 0.96 |
| 3-5 times | 1 (1.9) | 72 (0.8) | 2.39 (0.32-17.65) | 0.39 | 2.98 (0.40-22.10) | 0.29 |
| Antibiotics | 30 (51.7) | 3788 (39.7) | 1.62 (0.97-2.72) | 0.07 | 1.71 (1.01-2.90) | 0.04 |
| Bacterial infection | 31 (50.0) | 4030 (38.4) | 1.60 (0.97-2.64) | 0.06 | 1.68 (1.01-2.79) | 0.04 |
| Viral infection | 64 (98.5) | 10654 (98.0) | 1.32 (0.18-9.57) | 0.78 | 1.22 (0.17-8.89) | 0.84 |
| 1 to 3 years |  |  |  |  |  |  |
| Common cold |  |  |  |  |  |  |
| 1-2 times | 10 (20.8) | 968 (11.2) | 1.92 (0.89-4.14) | 0.10 | 1.74 (0.78-3.89) | 0.17 |
| 3-5 times | 19 (39.6) | 4103 (47.6) | 0.86 (0.46-1.63) | 0.64 | 0.84 (0.44-1.60) | 0.59 |
| 6 or more times | 19 (39.6) | 3530 (41.0) | 0.52 (0.24-1.12) | 0.09 | 0.57 (0.26-1.28) | 0.18 |
| Tonsillitis |  |  |  |  |  |  |
| 1-2 times | 8 (19.0) | 1142 (14.7) | 1.34 (0.62-2.89) | 0.46 | 1.43 (0.66-3.12) | 0.37 |
| Otitis media |  |  |  |  |  |  |
| 1-2 times | 19 (41.3) | 3060 (37.9) | 1.05 (0.57-1.93) | 0.87 | 1.01 (0.54-1.90) | 0.97 |
| 3-5 times | 4 (8.7) | 839 (10.4) | 0.81 (0.28-2.34) | 0.69 | 0.79 (0.27-2.30) | 0.66 |
| 6 or more times | 0 (0.0) | 285 (3.5) | na | 0.99 | na | 0.99 |
| Pneumonia |  |  |  |  |  |  |
| 1-2 times | 4 (9.5) | 556 (7.1) | 1.37 (0.49-3.86) | 0.55 | 1.39 (0.49-3.92) | 0.54 |
| Influenza |  |  |  |  |  |  |
| 1-2 times | 14 (32.6) | 2727 (35.2) | 0.91 (0.48-1.75) | 0.79 | 0.84 (0.43-1.65) | 0.62 |
| 3-5 times | 3 (7.0) | 342 (4.4) | 1.56 (0.47-5.18) | 0.47 | 1.57 (0.47-5.24) | 0.47 |
| Gastroenteritis | 37 (78.7) | 6062 (73.4) | 1.34 (0.67-2.70) | 0.41 | 1.45 (0.69-3.01) | 0.33 |
| 1-2 times | 27 (57.4) | 5028 (60.9) | 1.18 (0.57-2.45) | 0.65 | 1.31 (0.61-2.80) | 0.48 |
| 3-5 times | 5 (10.2) | 956 (11.6) | 1.85 (0.73-4.69) | 0.2 | 1.79 (0.66-4.83) | 0.25 |
| 6 or more times | 2 (4.3) | 65 (0.8) | 6.79 (1.46-31.59) | 0.02 | 8.30 (1.74-39.50) | 0.008 |
| Antibiotics | 35 (70.0) | 5783 (66.6) | 1.17 (0.64-2.15) | 0.61 | 1.19 (0.64-2.23) | 0.58 |
| 1-2 times | 28 (57.1) | 3890 (45.8) | 1.52 (0.81-2.85) | 0.19 | 1.55 (0.81-2.97) | 0.18 |
| 3-5 times | 5 (10.2) | 1162 (13.7) | 0.91 (0.33-2.50) | 0.85 | 0.90 (0.32-2.52) | 0.84 |
| 6 or more times | 1 (2.0) | 278 (3.3) | 0.76 (0.10-5.76) | 0.79 | 0.71 (0.09-5.48) | 0.75 |
| Bacterial infection | 35 (74.5) | 5814 (69.5) | 1.28 (0.66-2.47) | 0.46 | 1.34 (0.68-2.66) | 0.4 |
| Viral infection | 43 (87.8) | 7170 (85.3) | 1.24 (0.53-2.91) | 0.63 | 1.15 (0.48-2.71) | 0.76 |
| 3 to 5 years |  |  |  |  |  |  |
| Common cold |  |  |  |  |  |  |
| 1-2 times | 6 (14.6) | 511 (7.1) | 2.93 (1.12-7.65) | 0.03 | 2.90 (1.10-7.67) | 0.03 |
| 3-5 times | 21 (51.2) | 3218 (44.5) | 1.63 (0.83-3.21) | 0.16 | 1.46 (0.73-2.93) | 0.29 |
| 6 or more times | 14 (34.1) | 3491 (48.3) | 0.34 (0.13-0.89) | 0.03 | 0.35 (0.13-0.91) | 0.03 |
| Tonsillitis |  |  |  |  |  |  |
| 1-2 times | 8 (22.9) | 1378 (19.9) | 1.13 (0.51-2.48) | 0.77 | 1.09 (0.47-2.52) | 0.85 |
| Otitis media |  |  |  |  |  |  |
| 1-2 times | 8 (22.2) | 2533 (36.3) | 0.47 (0.21-1.04) | 0.06 | 0.44 (0.19-1.04) | 0.06 |
| 3-5 times | 2 (5.6) | 711 (10.2) | 0.42 (0.10-1.77) | 0.34 | 0.40 (0.09-1.70) | 0.21 |
| 6 or more times | 2 (5.6) | 189 (2.7) | 1.57 (0.37-6.68) | 0.54 | 1.45 (0.33-6.30) | 0.62 |
| Pneumonia |  |  |  |  |  |  |
| 1-2 times | 4 (11.4) | 360 (5.2) | 2.34 (0.82-6.67) | 0.11 | 2.17 (0.75-6.31) | 0.16 |
| Influenza |  |  |  |  |  |  |
| 1-2 times | 14 (36.8) | 2720 (39.7) | 0.98 (0.49-1.96) | 0.96 | 0.96 (0.47-1.97) | 0.91 |
| 3-5 times | 5 (13.2) | 459 (6.7) | 2.08 (0.77-5.59) | 0.15 | 2.25 (0.82-6.16) | 0.12 |
| Gastroenteritis | 39 (92.9) | 6352 (88.2) | 1.74 (0.54-5.65) | 0.36 | 2.54 (0.61-10.58) | 0.2 |
| 1-2 times | 26 (63.4) | 4390 (61.8) | 2.03 (0.55-9.73) | 0.26 | 4.32 (0.58-32.01) | 0.15 |
| 3-5 times | 13 (31.7) | 1768 (24.9) | 2.86 (0.64-12.71) | 0.17 | 5.59 (0.73-42.89) | 0.1 |
| 6 or more times | 0 (0.0) | 163 (2.3) | na | 0.99 | na | 0.99 |
| Antibiotics | 28 (66.7) | 5197 (71.1) | 0.81 (0.43-1.55) | 0.53 | 0.87 (0.45-1.70) | 0.69 |
| 1-2 times | 18 (43.9) | 3387 (47.5) | 0.77 (0.39-1.52) | 0.46 | 0.79 (0.39-1.58) | 0.5 |
| 3-5 times | 6 (14.6) | 1121 (15.7) | 0.78 (0.30-2.00) | 0.6 | 0.83 (0.32-2.15) | 0.7 |
| 6 or more times | 1 (2.4) | 290 (4.1) | 0.50 (0.07-3.80) | 0.51 | 0.49 (0.06-3.78) | 0.5 |
| Bacterial infection | 27 (69.2) | 5240 (73.0) | 0.83 (0.42-1.64) | 0.59 | 0.78 (0.39-1.57) | 0.49 |
| Viral infection | 40 (95.2) | 6582 (91.4) | 1.88 (0.45-7.80) | 0.38 | 3.54 (0.48-25.83) | 0.21 |
| National Patient Register |  |  |  |  |  |  |
| Respiratory Tract Infection | 15 (15.6) | 2481 (15.3) | 1.03 (0.59-1.79) | 0.92 | 0.91 (0.41-2.05) | 0.83 |
| 1-2 times | 15 (15.6) | 2378 (14.6) | 1.07 (0.62-1.87) | 0.8 | 0.96 (0.43-2.14) | 0.91 |
| 3 or more times | 0 (0.0) | 103 (0.6) | na | 0.99 | na | 0.99 |
| Gastroenteritis | 4 (4.2) | 219 (1.3) | 3.18 (1.16-8.74) | 0.03* | 2.93 (0.69-12.51) | 0.15 |
| Urinary Tract Infection | 2 (2.1) | 229 (1.4) | 1.49 (0.37-6.08) | 0.58 | 1.90 (0.26-14.03) | 0.53 |
| Unspecified infection | 8 (8.3) | 929 (5.7) | 1.50 (0.73-3.10) | 0.27 | 2.82 (1.25-6.35) | 0.01 |
| Total infection | 22 (22.9) | 3398 (20.9) | 1.13 (0.70-1.81) | 0.63 | 1.34 (0.71-2.54) | 0.37 |
| 1-2 times | 19 (19.8) | 3126 (19.2) | 1.06 (0.64-1.75) | 0.83 | 1.33 (0.69-2.57) | 0.4 |
| 3 or more times | 3 (3.1) | 272 (1.7) | 1.92 (0.60-6.12) | 0.27 | 1.52 (0.21-11.24) | 0.68 |
| Bacterial infection | 8 (8.3) | 1442 (8.9) | 0.93 (0.45-1.93) | 0.85 | 1.00 (0.36-2.80) | 0.99 |
| 1-2 times | 7 (7.3) | 1414 (8.7) | 0.83 (0.39-1.80) | 0.64 | 1.02 (0.36-2.85) | 0.98 |
| 3 or more times | 1 (1.0) | 28 (0.2) | 6.01 (0.81-44.68) | 0.08 | 6.48 (0.87-48.17) | 0.07 |
| Viral infection | 19 (19.8) | 2421 (14.9) | 1.41 (0.85-2.33) | 0.18 | 1.78 (0.92-3.44) | 0.09 |
| 1-2 times | 19 (19.8) | 2331 (14.3) | 1.47 (0.89-2.43) | 0.14 | 1.84 (0.95-3.56) | 0.07 |

*^a^Adjusted OR and adjusted p-values are calculated in a logistic regression analysis with sex, family history of type 1 diabetes and maternal education at birth. Bolded p-values are considered statistically significant after Benjamini & Hochberg correction for multiple comparisons.*

**Supplemental table 6: Males**

|  | Type 1 diabetes  n (%) | Reference group  n (%) | OR  (95 % CI) | p-value | Adj. OR^a^  (95 % CI) | Adj.  p-value^a^ |
| --- | --- | --- | --- | --- | --- | --- |
| **Total** | 94 (56.0) | 8390 (51.8) |  |  |  |  |
| **1-12 months** |  |  |  |  |  |  |
| Common cold |  |  |  |  |  |  |
| 1-2 times | 19 (29.7) | 1787 (33.3) | 0.86 (0.11-6.51) | 0.89 | 0.90 (0.12-6.91) | 0.92 |
| 3-5 times | 35 (54.7) | 2600 (48.4) | 1.09 (0.15-8.06) | 0.96 | 1.07 (0.14-6.91) | 0.97 |
| 6 or more times | 9 (14.1) | 905 (16.8) | 0.81 (0.10-6.44) | 0.92 | 0.79 (0.10-6.40) | 0.9 |
| Otitis media | 19 (32.2) | 1429 (28.2) | 1.21 (0.70-2.10) | 0.5 | 1.26 (0.72-2.20) | 0.42 |
| Pneumonia | 3 (5.4) | 353 (7.3) | 0.72 (0.22-2.30) | 0.58 | 0.73 (0.22-2.36) | 0.6 |
| Influenza |  |  |  |  |  |  |
| 1-2 times | 7 (12.5) | 598 (13.0) | 0.96 (0.43-2.14) | 0.93 | 1.03 (0.46-2.29) | 0.95 |
| 3-5 times | 1 (1.8) | 33 (0.7) | 2.49 (0.33-18.61) | 0.37 | 1.49 (0.18-12.53) | 0.71 |
| Gastroenteritis | 19 (31.1) | 1593 (29.9) | 1.06 (0.61-1.83) | 0.84 | 1.07 (0.62-1.86) | 0.81 |
| 1-2 times | 16 (29.1) | 1454 (30.4) | 0.95 (0.53-1.72) | 0.88 | 0.95 (0.53-1.73) | 0.87 |
| 3-5 times | 1 (1.8) | 36 (0.8) | 2.41 (0.32-18.03) | 0.39 | 3.16 (0.42-23.80) | 0.27 |
| Antibiotics | 31 (50.8) | 2083 (42.0) | 1.43 (0.86-2.36) | 0.17 | 1.50 (0.90-2.50) | 0.12 |
| Bacterial infection | 33 (51.6) | 2231 (40.9) | 1.54 (0.94-2.52) | 0.09 | 1.63 (0.98-2.68) | 0.06 |
| Viral infection | 66 (98.5) | 5531 (98.1) | 1.28 (0.18-9.29) | 0.81 | 1.27 (0.17-9.32) | 0.82 |
| **1 to 3 years** |  |  |  |  |  |  |
| Common cold |  |  |  |  |  |  |
| 1-2 times | 10 (17.2) | 466 (10.5) | 1.70 (0.81-3.57) | 0.17 | 1.74 (0.82-3.71) | 0.15 |
| 3-5 times | 24 (41.4) | 2082 (46.7) | 0.91 (0.52-1.61) | 0.75 | 0.90 (0.51-1.62) | 0.73 |
| 6 or more times | 24 (41.4) | 1896 (42.6) | 0.59 (0.28-1.24) | 0.17 | 0.57 (0.27-1.22) | 0.14 |
| Tonsillitis |  |  |  |  |  |  |
| 1-2 times | 7 (13.7) | 622 (15.6) | 0.84 (0.38-1.88) | 0.68 | 0.92 (0.41-2.06) | 0.83 |
| Otitis media |  |  |  |  |  |  |
| 1-2 times | 25 (43.1) | 1639 (39.3) | 1.15 (0.66-2.02) | 0.61 | 1.26 (0.71-2.22) | 0.43 |
| 3-5 times | 7 (12.1) | 477 (11.4) | 1.11 (0.48-2.59) | 0.81 | 1.12 (0.47-2.63) | 0.8 |
| 6 or more times | 1 (1.7) | 157 (3.8) | 0.48 (0.07-3.58) | 0.48 | 0.42 (0.06-3.16) | 0.4 |
| Pneumonia |  |  |  |  |  |  |
| 1-2 times | 6 (11.5) | 287 (7.1) | 1.69 (0.72-3.99) | 0.23 | 1.63 (0.68-3.89) | 0.27 |
| Influenza |  |  |  |  |  |  |
| 1-2 times | 17 (33.3) | 1396 (35.1) | 0.87 (0.48-1.56) | 0.63 | 0.86 (0.48-1.57) | 0.63 |
| 3-5 times | 1 (2.0) | 202 (5.1) | 0.35 (0.05-2.58) | 0.3 | 0.38 (0.05-2.78) | 0.34 |
| Gastroenteritis | 43 (75.4) | 3133 (73.6) | 1.10 (0.60-2.02) | 0.75 | 1.23 (0.66-2.31) | 0.52 |
| 1-2 times | 29 (51.8) | 2591 (60.9) | 0.90 (0.48-1.72) | 0.75 | 1.00 (0.51-1.94) | 0.99 |
| 3-5 times | 10 (17.9) | 500 (11.7) | 1.61 (0.71-3.66) | 0.25 | 1.84 (0.80-4.27) | 0.15 |
| 6 or more times | 3 (5.4) | 36 (0.8) | 6.72 (1.85-24.42) | **0.004** | 8.88 (2.39-32.96) | **0.001** |
| Antibiotics | 43 (72.9) | 3083 (68.7) | 1.23 (0.69-2.18) | 0.49 | 1.36 (0.75-2.48) | 0.31 |
| 1-2 times | 32 (55.2) | 2061 (47.0) | 1.39 (0.77-2.51) | 0.28 | 1.57 (0.85-2.89) | 0.15 |
| 3-5 times | 7 (12.1) | 651 (14.9) | 0.96 (0.40-2.33) | 0.93 | 0.97 (0.39-2.38) | 0.94 |
| 6 or more times | 2 (3.4) | 152 (3.5) | 1.18 (0.27-5.14) | 0.83 | 1.10 (0.25-4.89) | 0.9 |
| Bacterial infection | 44 (77.2) | 3088 (71.5) | 1.35 (0.72-2.51) | 0.35 | 1.53 (0.80-2.91) | 0.2 |
| Viral infection | 50 (86.2) | 3719 (85.7) | 1.05 (0.49-2.22) | 0.91 | 1.00 (0.47-2.12) | 0.99 |
| **3 to 5 years** |  |  |  |  |  |  |
| Common cold |  |  |  |  |  |  |
| 1-2 times | 5 (11.9) | 264 (7.0) | 2.37 (0.86-6.59) | 0.10 | 2.39 (0.85-6.69) | 0.10 |
| 3-5 times | 22 (52.4) | 1621 (43.0) | 1.70 (0.88-3.29) | 0.11 | 1.63 (0.84-3.17) | 0.15 |
| 6 or more times | 15 (35.7) | 1880 (49.9) | 0.42 (0.15-1.17) | 0.09 | 0.42 (0.15-1.17) | 0.10 |
| Tonsillitis |  |  |  |  |  |  |
| 1-2 times | 8 (20.5) | 744 (20.6) | 0.96 (0.44-2.11) | 0.93 | 0.95 (0.43-2.10) | 0.91 |
| Otitis media |  |  |  |  |  |  |
| 1-2 times | 12 (30.0) | 1322 (36.3) | 0.68 (0.34-1.37) | 0.28 | 0.74 (0.37-1.49) | 0.4 |
| 3-5 times | 2 (5.0) | 406 (11.1) | 0.37 (0.09-1.57) | 0.18 | 0.35 (0.08-1.52) | 0.16 |
| 6 or more times | 2 (5.0) | 113 (3.1) | 1.33 (0.31-5.69) | 0.7 | 1.27 (0.29-5.50) | 0.75 |
| Pneumonia |  |  |  |  |  |  |
| 1-2 times | 4 (10.5) | 182 (5.1) | 2.20 (0.77-6.27) | 0.14 | 1.78 (0.67-4.72) | 0.24 |
| Influenza |  |  |  |  |  |  |
| 1-2 times | 12 (30.0) | 1429 (40.1) | 0.71 (0.35-1.43) | 0.33 | 0.67 (0.34-1.42) | 0.32 |
| 3-5 times | 6 (15.0) | 257 (7.2) | 1.96 (0.79-4.89) | 0.15 | 2.14 (0.85-5.37) | 0.11 |
| Gastroenteritis | 40 (93.0) | 3311 (88.3) | 1.77 (0.55-5.75) | 0.34 | 1.90 (0.58-6.21) | 0.29 |
| 1-2 times | 24 (55.8) | 2268 (61.2) | 1.44 (0.43-4.79) | 0.56 | 1.50 (0.45-5.02) | 0.51 |
| 3-5 times | 16 (37.2) | 941 (25.4) | 2.31 (0.67-7.96) | 0.19 | 2.42 (0.70-8.39) | 0.16 |
| 6 or more times | 0 (0.0) | 88 (2.4) | na | 0.99 | na | 0.99 |
| Antibiotics | 30 (69.8) | 2743 (72.0) | 0.90 (0.47-1.72) | 0.74 | 0.94 (0.49-1.82) | 0.86 |
| 1-2 times | 17 (41.5) | 1769 (47.6) | 0.75 (0.37-1.50) | 0.41 | 0.77 (0.38-1.55) | 0.46 |
| 3-5 times | 7 (17.1) | 607 (16.3) | 0.90 (0.36-2.21) | 0.81 | 0.86 (0.35-2.12) | 0.74 |
| 6 or more times | 2 (4.9) | 171 (4.6) | 0.91 (0.21-4.01) | 0.9 | 0.96 (0.22-4.26) | 0.95 |
| Bacterial infection | 29 (70.7) | 2759 (73.8) | 0.86 (0.44-1.69) | 0.66 | 0.90 (0.45-1.77) | 0.76 |
| Viral infection | 42 (97.7) | 3439 (91.8) | 3.77 (0.52-27.51) | 0.19 | 3.90 (0.53-28.53) | 0.18 |
| **National Patient Register** |  |  |  |  |  |  |
| Respiratory Tract Infection | 14 (14.9) | 1476 (17.6) | 0.82 (0.46-1.45) | 0.5 | 0.76 (0.36-1.61) | 0.47 |
| 1-2 times | 13 (13.8) | 1406 (16.8) | 0.80 (0.44-1.44) | 0.46 | 0.80 (0.38-1.70) | 0.56 |
| 3 or more times | 1 (1.1) | 70 (0.8) | 1.24 (0.17-9.00) | 0.84 | 1.12 (0.15-8.26) | 0.91 |
| Gastroenteritis | 4 (4.3) | 123 (1.5) | 2.99 (1.08-8.26) | 0.04 | 2.34 (0.54-10.07) | 0.25 |
| Urinary Tract Infection | 2 (2.1) | 76 (0.9) | 2.38 (0.58-9.83) | 0.23 | 5.01 (1.17-21.44) | 0.03 |
| Unspecified infection | 9 (9.6) | 503 (6.0) | 1.66 (0.83-3.32) | 0.15 | 2.48 (1.15-5.33) | 0.02 |
| Total infection | 22 (23.4) | 1921 (22.9) | 1.03 (0.64-1.66) | 0.91 | 1.05 (0.57-1.93) | 0.88 |
| 1-2 times | 18 (19.1) | 1760 (21.0) | 0.92 (0.55-1.55) | 0.75 | 0.97 (0.51-1.84) | 0.91 |
| 3 or more times | 4 (4.3) | 161 (1.9) | 2.23 (0.81-6.19) | 0.12 | 2.15 (0.50-9.17) | 0.3 |
| Bacterial infection | 9 (9.6) | 755 (9.0) | 1.07 (0.54-2.14) | 0.85 | 1.26 (0.53-2.97) | 0.6 |
| 1-2 times | 9 (9.6) | 740 (8.8) | 1.09 (0.55-2.18) | 0.8 | 1.28 (0.54-3.02) | 0.57 |
| Viral infection | 20 (21.3) | 1428 (17.0) | 1.32 (0.80-2.17) | 0.28 | 1.37 (0.74-2.57) | 0.32 |
| 1-2 times | 20 (21.3) | 1372 (16.4) | 1.37 (0.83-2.26) | 0.21 | 1.43 (0.76-2.67) | 0.26 |

*^a^Adjusted OR and adjusted p-values are calculated in a logistic regression analysis with sex, family history of type 1 diabetes and maternal education at birth. Bolded p-values are considered statistically significant after Benjamini & Hochberg correction for multiple comparisons.*

**Supplemental table 7: Females**

|  | Type 1 diabetes  n (%) | Reference group  n (%) | OR  (95 % CI) | p-value | Adj. OR^a^  (95 % CI) | Adj.  p-value^a^ |
| --- | --- | --- | --- | --- | --- | --- |
| Total | 74 (44.0) | 7808 (48.2) |  |  |  |  |
| 1-12 months |  |  |  |  |  |  |
| Common cold |  |  |  |  |  |  |
| 1-2 times | 13 (34.2) | 1727 (34.5) | 1.00 (0.38-2.64) | 0.99 | 1.01 (0.38-2.67) | 0.99 |
| 3-5 times | 19 (50.0) | 2385 (47.7) | 1.06 (0.42-2.66) | 0.91 | 1.06 (0.42-2.68) | 0.90 |
| 6 or more times | 6 (15.8) | 796 (15.9) | 1.00 (0.38-2.64) | 0.99 | 0.99 (0.37-2.64) | 0.99 |
| Otitis media | 8 (20.5) | 1152 (24.5) | 0.80 (0.37-1.74) | 0.57 | 0.81 (0.37-1.77) | 0.59 |
| Pneumonia | 1 (2.6) | 258 (5.8) | 0.44 (0.06-3.24) | 0.42 | 0.43 (0.06-3.17) | 0.41 |
| Influenza |  |  |  |  |  |  |
| 1-2 times | 5 (15.2) | 524 (12.4) | 1.26 (0.48-3.27) | 0.64 | 1.33 (0.51-3.48) | 0.56 |
| 3-5 times | 0 (0.0) | 25 (0.6) | na | 0.99 | na | 0.99 |
| Gastroenteritis | 16 (40.0) | 1417 (28.8) | 1.65 (0.87-3.11) | 0.12 | 1.70 (0.90-3.23) | 0.1 |
| 1-2 times | 13 (37.1) | 1304 (29.6) | 1.46 (0.73-2.92) | 0.29 | 1.52 (0.76-3.07) | 0.24 |
| 3-5 times | 1 (2.9) | 36 (0.8) | 4.06 (0.53-30.97) | 0.18 | 5.10 (0.66-39.29) | 0.12 |
| Antibiotics | 13 (40.6) | 1705 (37.3) | 1.15 (0.57-2.33) | 0.7 | 1.22 (0.60-2.50) | 0.59 |
| Bacterial infection | 13 (33.3) | 1799 (35.8) | 0.90 (0.46-1.75) | 0.75 | 0.93 (0.47-1.82) | 0.82 |
| Viral infection | 40 (100) | 5118 (97.8) | na | 0.99 | na | 0.99 |
| 1 to 3 years |  |  |  |  |  |  |
| Common cold |  |  |  |  |  |  |
| 1-2 times | 6 (20.7) | 499 (12.0) | 1.96 (0.71-5.41) | 0.19 | 1.27 (0.40-3.96) | 0.69 |
| 3-5 times | 13 (44.8) | 2014 (48.6) | 1.05 (0.46-2.40) | 0.91 | 1.02 (0.43-2.43) | 0.96 |
| 6 or more times | 10 (34.5) | 1627 (39.2) | 0.51 (0.19-1.41) | 0.20 | 0.79 (0.25-2.47) | 0.69 |
| Tonsillitis |  |  |  |  |  |  |
| 1-2 times | 4 (14.8) | 519 (13.8) | 1.07 (0.37-3.09) | 0.91 | 1.20 (0.40-3.55) | 0.75 |
| Otitis media |  |  |  |  |  |  |
| 1-2 times | 11 (37.9) | 1412 (36.3) | 0.91 (0.43-1.96) | 0.82 | 0.89 (0.40-1.99) | 0.78 |
| 3-5 times | 1 (3.4) | 360 (9.2) | 0.33 (0.04-2.46) | 0.28 | 0.38 (0.05-2.92) | 0.35 |
| 6 or more times | 0 (0.0) | 128 (3.3) | na | 0.99 | na | 0.99 |
| Pneumonia |  |  |  |  |  |  |
| 1-2 times | 4 (14.3) | 268 (7.1) | 2.19 (0.75-6.34) | 0.15 | 1.78 (0.52-6.10) | 0.36 |
| Influenza |  |  |  |  |  |  |
| 1-2 times | 9 (33.3) | 1327 (35.3) | 0.96 (0.43-2.19) | 0.93 | 0.91 (0.38-2.15) | 0.82 |
| 3-5 times | 2 (7.4) | 138 (3.7) | 2.06 (0.47-9.05) | 0.34 | 2.70 (0.60-12.17) | 0.2 |
| Gastroenteritis | 20 (74.1) | 2915 (73.2) | 1.05 (0.44-2.48) | 0.92 | 0.89 (0.37-2.17) | 0.8 |
| 1-2 times | 12 (44.4) | 2424 (60.9) | 0.76 (0.30-1.93) | 0.56 | 0.74 (0.29-1.91) | 0.53 |
| 3-5 times | 7 (25.9) | 455 (11.4) | 2.36 (0.82-6.76) | 0.11 | 1.51 (0.47-4.91) | 0.49 |
| 6 or more times | 1 (3.7) | 29 (0.7) | 5.29 (0.63-44.37) | 0.13 | 6.58 (0.77-56.09) | 0.09 |
| Antibiotics | 17 (56.7) | 2687 (64.3) | 0.73 (0.35-1.50) | 0.39 | 0.67 (0.32-1.43) | 0.3 |
| 1-2 times | 12 (40.0) | 1819 (44.5) | 0.72 (0.34-1.54) | 0.4 | 0.70 (0.32-1.54) | 0.37 |
| 3-5 times | 3 (10.0) | 508 (12.4) | 0.65 (0.19-2.24) | 0.49 | 0.47 (0.11-2.10) | 0.32 |
| 6 or more times | 0 (0.0) | 126 (3.1) | na | 0.99 | na | 0.99 |
| Bacterial infection | 16 (55.2) | 2714 (67.3) | 0.60 (0.29-1.25) | 0.17 | 0.58 (0.27-1.26) | 0.17 |
| Viral infection | 26 (89.7) | 3435 (84.9) | 1.55 (0.47-5.13) | 0.48 | 1.52 (0.45-5.14) | 0.5 |
| 3 to 5 years |  |  |  |  |  |  |
| Common cold |  |  |  |  |  |  |
| 1-2 times | 2 (7.4) | 246 (7.1) | 1.31 (0.29-6.01) | 0.73 | 1.12 (0.24-5.30) | 0.88 |
| 3-5 times | 15 (55.6) | 1596 (46.2) | 1.51 (0.68-3.38) | 0.31 | 1.05 (0.45-2.48) | 0.91 |
| 6 or more times | 10 (37.0) | 1611 (46.6) | 0.76 (0.17-3.51) | 0.73 | 0.89 (0.19-4.20) | 0.89 |
| Tonsillitis |  |  |  |  |  |  |
| 1-2 times | 3 (12.5) | 634 (19.2) | 0.57 (0.17-1.92) | 0.41 | 0.53 (0.12-2.34) | 0.4 |
| Otitis media |  |  |  |  |  |  |
| 1-2 times | 4 (16.7) | 1211 (36.2) | 0.30 (0.10-0.90) | 0.03 | 0.14 (0.03-0.63) | 0.01 |
| 3-5 times | 0 (0.0) | 305 (9.1) | na | 0.99 | na | 0.99 |
| 6 or more times | 1 (4.2) | 76 (2.3) | 1.21 (0.16-9.17) | 0.85 | 1.41 (0.18-11.11) | 0.75 |
| Pneumonia |  |  |  |  |  |  |
| 1-2 times | 1 (4.0) | 178 (5.4) | 0.76 (0.10-5.69) | 0.79 | 0.61 (0.08-4.73) | 0.63 |
| 3-5 times | 1 (4.0) | 13 (0.4) | 10.46 (1.31-83.26) | 0.03 | 15.23 (1.88-123.59) | 0.01 |
| Influenza |  |  |  |  |  |  |
| 1-2 times | 12 (46.2) | 1290 (39.3) | 1.50 (0.66-3.41) | 0.34 | 1.87 (0.75-4.65) | 0.18 |
| 3-5 times | 2 (7.7) | 202 (6.1) | 1.59 (0.35-7.24) | 0.55 | 1.97 (0.39-10.01) | 0.41 |
| Gastroenteritis | 25 (92.6) | 3039 (88.1) | 1.69 (0.40-7.16) | 0.48 | 2.62 (0.35-19.69) | 0.35 |
| 1-2 times | 16 (61.5) | 2120 (62.5) | 2.80 (0.37-21.18) | 0.32 | 2.04 (0.26-15.70) | 0.5 |
| 3-5 times | 8 (30.8) | 827 (24.4) | 3.59 (0.45-28.80) | 0.23 | 3.03 (0.37-24.645) | 0.3 |
| 6 or more times | 1 (3.8) | 75 (2.2) | 4.95 (0.30-79.97) | 0.26 | 4.95 (0.30-81.34) | 0.26 |
| Antibiotics | 18 (66.7) | 2453 (70.0) | 0.86 (0.38-1.91) | 0.71 | 0.95 (0.40-2.26) | 0.9 |
| 1-2 times | 13 (48.1) | 1617 (47.4) | 0.94 (0.41-2.14) | 0.88 | 0.90 (0.37-2.21) | 0.82 |
| 3-5 times | 4 (14.8) | 514 (15.1) | 0.91 (0.28-2.90) | 0.87 | 1.23 (0.37-4.11) | 0.74 |
| 6 or more times | 0 (0.0) | 119 (3.5) | na | 0.99 | na | 0.99 |
| Bacterial infection | 17 (65.4) | 2481 (72.2) | 0.73 (0.32-1.63) | 0.44 | 0.61 (0.26-1.45) | 0.26 |
| Viral infection | 25 (92.6) | 3141 (91.0) | 1.23 (0.29-5.23) | 0.78 | 2.01 (0.27-15.19) | 0.5 |
| National Patient Register |  |  |  |  |  |  |
| Respiratory Tract Infection | 10 (13.5) | 1005 (12.9) | 1.06 (0.54-2.07) | 0.87 | 0.26 (0.04-1.92) | 0.17 |
| 1-2 times | 10 (13.5) | 972 (12.4) | 1.09 (0.56-2.14) | 0.79 | 0.26 (0.04-1.96) | 0.19 |
| Gastroenteritis | 0 (0) | 96 (1.2) | na | 0.99 | na | 0.99 |
| Urinary tract infection | 2 (2.7) | 153 (2.0) | 1.39 (0.34-5.72) | 0.65 | 1.58 (0.38-6.50) | 0.53 |
| Unspecified infection | 3 (4.1) | 426 (5.5) | 0.73 (0.23-2.33) | 0.6 | 0.60 (0.08-4.53) | 0.62 |
| Total infection | 11 (14.9) | 1477 (18.9) | 0.75 (0.39-1.42) | 0.38 | 0.32 (0.07-1.34) | 0.12 |
| 1-2 times | 9 (12.2) | 1366 (17.5) | 0.66 (0.33-1.34) | 0.25 | 0.33 (0.08-1.41) | 0.14 |
| 3 or more times | 2 (2.7) | 111 (1.4) | 1.81 (0.44-7.49 | 0.41 | 2.05 (0.50-8.52) | 0.32 |
| Bacterial infection | 4 (5.4) | 687 (8.8) | 0.59 (0.22-1.63) | 0.31 | 0.63 (0.23-1.74) | 0.38 |
| 1-2 times | 3 (4.1) | 674 (8.6) | 0.45 (0.14-1.44) | 0.18 | 0.48 (0.15-1.54) | 0.22 |
| Viral infection | 10 (13.5) | 993 (12.7) | 1.07 (0.55-2.10) | 0.84 | 0.48 (0.11-2.04) | 0.32 |
| 1-2 times | 9 (12.2) | 959 (12.3) | 3.13 (0.42-23.23) | 0.26 | 0.49 (0.11-2.08) | 0.33 |

*^a^Adjusted OR and adjusted p-values are calculated in a logistic regression analysis with sex, family history of type 1 diabetes and maternal education at birth. Bolded p-values are considered statistically significant after Benjamini & Hochberg correction for multiple comparisons.*
